# Constrained Ordination Analysis with Enrichment of Bell-Shaped Response Functions

**DOI:** 10.1371/journal.pone.0154079

**Published:** 2016-04-21

**Authors:** Yingjie Zhang, Olivier Thas

**Affiliations:** 1 Department of Mathematical Modelling, Statistics and Bioinformatics, Ghent University, Coupure Links 653, Ghent 9000, Belgium; 2 National Institute for Applied Statistics Research Australia (NIASRA), School of Mathematics and Applied Statistics, University of Wollongong, Northfields Ave, Wollongong NSW 2522, Australia; Indiana University Bloomington, UNITED STATES

## Abstract

Constrained ordination methods aims at finding an environmental gradient along which the species abundances are maximally separated. The species response functions, which describe the expected abundance as a function of the environmental score, are according to the ecological fundamental niche theory only meaningful if they are bell-shaped. Many classical model-based ordination methods, however, use quadratic regression models without imposing the bell-shape and thus allowing for meaningless U-shaped response functions. The analysis output (e.g. a biplot) may therefore be potentially misleading and the conclusions are prone to errors. In this paper we present a log-likelihood ratio criterion with a penalisation term to enforce more bell-shaped response shapes. We report the results of a simulation study and apply our method to metagenomics data from microbial ecology.

## Introduction

Constrained or Canonical Correspondence Analysis (CCA) is a well established method among environmental ecologists to study the relation between species abundances and the environmental conditions. This method, which is nowadays looked at as a particular technique for Constrained Ordination Analysis (COA), was originally introduced by ter Braak [1] as an extension of the correspondence analysis method for exploring the dependence structure in a two-way contingency table or a species-by-sample abundance table; see, for example, Greenacre [2], ter Braak [3] and ter Braak and Šmilauer [4] for recent accounts.

In a CCA and COA the species abundances are regressed on environmental scores that result from linear combinations of the environmental conditions of the sampling locations. The linear combination is referred to as the environmental gradient. The coefficients defining the environmental gradient are the same for all species, but each species has its abundances described by another *response function* that relates the abundances to the environmental scores. The CCA method aims at finding the gradient that maximally separates the response functions. When the species-specific response functions are described by a Gaussian density function and the abundances are assumed to follow a Poisson distribution, a Constrained Gaussian Ordination (CGO) model arises. ter Braak [1] showed that his eigenvalue-based CCA can be considered as an approximation to the maximum likelihood solution of a CGO model. The approximation was studied in a simulation study by Johnson and Altman [5]; they concluded that the approximation is good when the species-specific tolerances are more or less equal (see later). While the eigenvalue-approach of CCA has the advantage of being computationally more efficient, the model-based likelihood method has the advantage of being more flexible as it allows for replacing the Poisson and Gaussian components with other functional forms, numerical instabilities notwithstanding.

**Fig 1:**
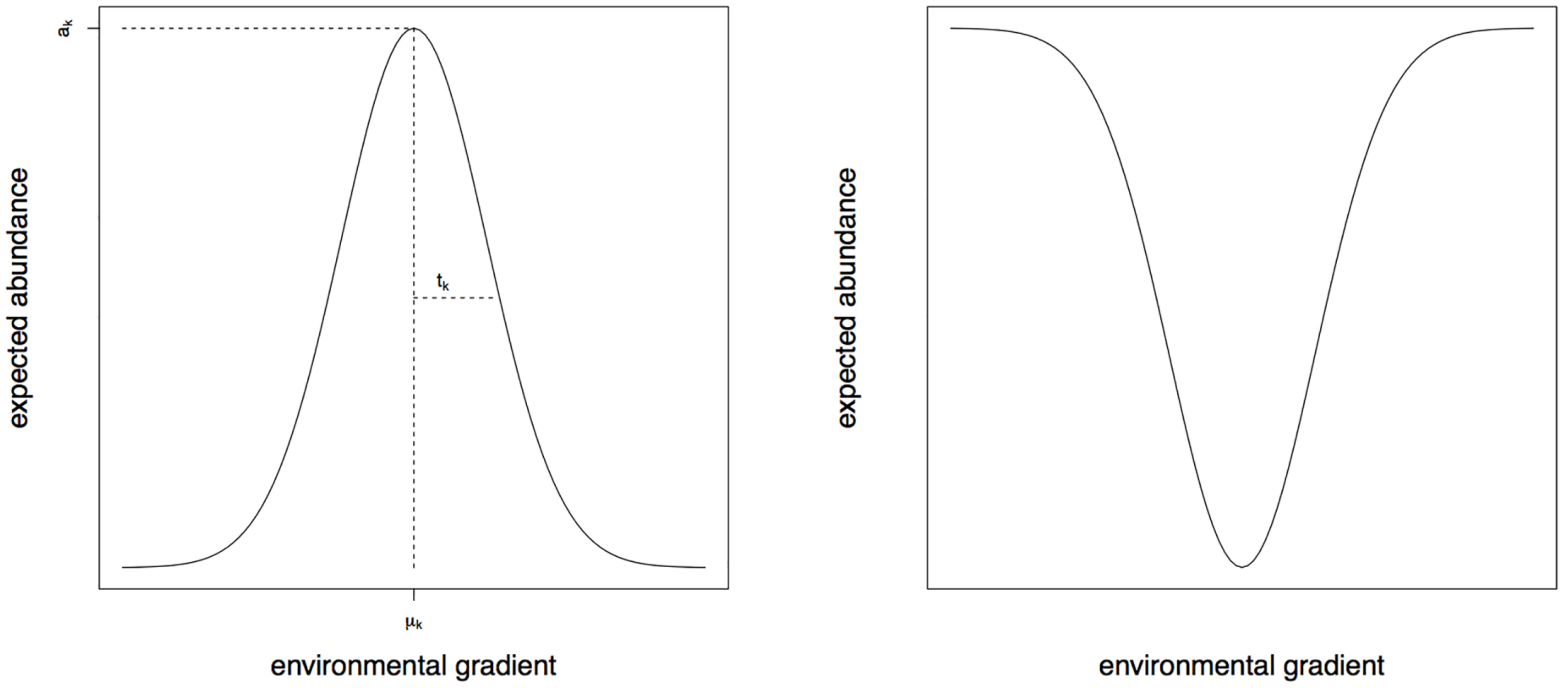
A Gaussian response curve (left) and a U-shaped response curve (right). The left panel also indicates the parameters *μ*_*k*_ (optimum), *a*_*k*_ (maximum) and *t*_*k*_ (tolerance).

The species response function is an important concept in community ecology and particularly for the quantitative analysis of ecological niches. Just like the CCA and CGO methods, the new method developed in this paper is appropriate for studying the *realised niches* or the *fundamental niches* of species. The latter can be defined as the range of environmental conditions under which the species can exists without interspecies competition or predation from other species [6]. Within a fundamental niche, the species response function has a single maximum that corresponds to the optimal environmental conditions for the existence of the species. When moving away from the optimal condition the abundances are expected to decrease; the relation is typically non-linear [6, 7]. Gauch et al. [8] developed a Gaussian ordination method to reveal the species-environment relation in which each species response function was assumed to be a unimodal bell-shaped curve, called a Gaussian curve; this is illustrated in the left panel of Fig 1. The response function is characterised by three parameters: the maximum *a*_*k*_, optimum *μ*_*k*_ and the tolerance *t*_*k*_. In 1986 ter Braak [1] introduced the CCA method which soon became the dominating ordination analysis method in community ecology. The method is based on Gaussian response curves with equal tolerances.

The *realised niche* of a species is a subset of the fundamental niche, and describes the environmental conditions under which the species can exists with interspecies competition and predation from other species. The symmetric unimodal response function has been criticised, particularly for studying the realised niches of a species. The competition among species may cause unimodal response functions of different skewness [9]. Many studies have focused on the use of more flexible species response curve. For example, Austin [10, 11] suggested that the *β*-function is a more realistic unimodal response function. However, computational difficulties in estimating the *β*-function parameters obstruct the application of *β*-functions in ordination analysis [12, 13]. For the same reasons Huisman et al. [14] proposed the HOF model which contains a set of submodels allowing different degrees of skewness and flatness. Note that the most complex HOF model has 5 parameters, causing the HOF model to suffer from similar computational obstacles as the *β*-function. Besides the technical difficulties, another reason that these more complex models are not very popular may be that bell-shaped curves are a realistic reflection of the fundamental niche theory and in many ecological studies the unimodal bell-shaped response functions show sufficient approximation to the data.

Some papers [15, 16] report attempts to embed ordination methods in classical statistical modelling frameworks, allowing for more flexibility in describing the response functions. For example, the work of Zhu et al. [15] enables modelling each individual species in the community with maximal flexibility, both parametrically and nonparametrically. They proposed a likelihood ratio statistic that measures the separation of species response functions. Finding the gradient along which the species display maximally separated response functions is obtained by maximising their likelihood ratio criterion. Their method will be referred to as Flexible Constrained Ordination Analysis (FCOA).

Despite the flexibility of the FCOA method, users should be careful because any type of species response curve could be fitted, even if it is ecologically meaningless. For instance, one could use the popular second order polynomial Poisson regression model for relating the expected abundance to the environmental scores, but without any constraints on the polynomial regression coefficients the fitted curve can be either bell-shaped (concave) or U-shaped (convex). This is illustrated in Fig 1. Consequently, the result may be misleading, for the U-shaped response functions are often ecologically meaningless, particularly in the niche theory. This raises the question whether the contributions made by these U-shaped functions to the likelihood ratio criterion should not be counted at all, as they may eventually lead to the wrong or suboptimal environmental gradient. The problem becomes even worse when observing that many ordination software implementations do not allow the user to assess the quality and relevance of the model fit. Instead the ordination results are typically summarised in a biplot which shows the environmental gradients and the estimated optimum parameters (*μ*_*k*_) for all species. However, when a species *μ*_*k*_ parameter comes from a U-shaped response function, it does not represent the optimal environmental conditions, but quite the opposite. From the biplot, conclusions are formulated about similarities/differences between species responses to environmental conditions, assuming that all species show ecologically meaningful bell-shaped response functions along the environmental gradients.

In this paper we propose a penalized maximum likelihood method in which the penalization forces many response functions to be bell-shaped. We refer to the new method as BECOA (Bell-shape Enriched COA). The method is empirically evaluated in a simulation study and it is applied to the microbial diversity data. The paper ends with a discussion and the formulation of conclusions.

## Bell-Shape Enriched Constrained Ordination Analysis

### Model-Based Constrained Ordination Analysis

Our method builds on the construction of Zhu et al. [15] for constrained ordination. First some notation is introduced. Let $x_{i}^{t}=\left(x_{i1},\ldots ,x_{ip}\right)$ denote the *p*-dimensional vector of environmental variables measured at sampling location *i* = 1, …, *n*. The vector with the abundances of species *k* = 1, …, *s* at the *n* locations is denoted by $Y_{k}^{t}=\left(Y_{1k},\ldots ,Y_{nk}\right)$. For a given coefficient vector *α*^*t*^ = (*α*_1_, …, *α*_*p*_), the environmental observations are transformed to univariate environmental scores given by *z*_*i*_ = ***α***^*t*^***x***_*i*_, *i* = 1, …, *n*. The abundance of species *k* at location *i* is assumed to be Poisson distributed, conditional on *z*_*i*_. In particular,

$$\begin{array}{c} Y_{ik}\left|x_{i}∼Y_{ik}\right|z_{i}∼\text{Poisson}\left(\lambda_{ik}\right), \end{array}$$

where λ_*ik*_ = E{*Y*_*ik*_∣***x***_*i*_;***β***_*k*_} = E{*Y*_*ik*_∣*z*_*i*_;***β***_*k*_} in which ***β***_*k*_ is a regression parameter vector. The probability mass function is denoted by *p*_*k*_(*y*∣*z*,***β***_*k*_). In the present context the Poisson mean function is referred to as the response function of species *k*, denoted by *f*_*k*_(*z*_*i*_;***β***_*k*_), or simply *f*_*k*_(*z*_*i*_).

If we assume that all species respond in the same way to the environment, the index *k* may be dropped from the response function, resulting in a common response function, say *f*(*z*_*i*_). The corresponding Poisson probability mass function is denoted by *p*(*y*∣*z*;***β***), and the ***β*** parameter may be estimated using data of all species simultaneously.

Zhu et al. [15] proposed an iterative scheme for the joint estimation of ***α*** and the ***β***_*k*_ parameters: (1) for an initial ***α*** the environmental variables are transformed to the *z* scores; (2) estimate the ***β***_*k*_ and ***β*** from the corresponding Poisson regression models; (3) estimate ***α*** by maximising the log-likelihood ratio (LLR) criterion

$$\begin{array}{c} \text{LLR}\left(\alpha \right)=\sum_{i=1}^{n}\sum_{k=1}^{s}\log\frac{p_{k}\left(y_{ik}|z_{i};\beta_{k}\right)}{p(y_{ik}|z_{i};\beta )} \end{array}$$ (1)

with the ***β*** and ***β***_*k*_ parameters replaced by their estimates from step (2); (4) repeat steps (2) and (3) until convergence. The rationale for the use of the LLR criterion is given later in this section.

The response function is often believed to be unimodal. A popular choice is the Gaussian response model, which borrows its name from its similarity to a Gaussian density function. Upon using the canonical log-link function of Poisson regression, the model can be written as

$$\begin{array}{c} \log f_{k}\left(z_{i}\right)=a_{k}-\frac{{\left(z_{i}-\mu_{k}\right)}^{2}}{2t_{k}^{2}} \end{array}$$ (2)

in which $\beta_{k}^{t}=\left(a_{k},\mu_{k},t_{k}\right)$ represents the species-specific parameters to be estimated from the data. Despite the convenient interpretation of the parameters, model formulation Eq (2) is nonlinear in the parameters and hence computationally less attractive. Model (2) is therefore often replaced by the log-linear model

$$\begin{array}{c} \log f_{k}\left(z_{i}\right)=\beta_{0k}+\beta_{1k}z_{i}+\beta_{2k}z_{i}^{2}=\beta_{k}^{t}w_{i}, \end{array}$$ (3)

with $\beta_{k}^{t}=\left(\beta_{0k},\beta_{1k},\beta_{2k}\right)$ and $w_{i}^{t}=\left(1,z_{i},z_{i}^{2}\right)$. For a given ***α***, this model allows for the use of standard GLM software for the estimation of the *β* parameters. However, with ${\hat{\beta }}_{k}^{t}=\left({\hat{\beta }}_{0k},{\hat{\beta }}_{1k},{\hat{\beta }}_{2k}\right)$ denoting the vector with the parameter estimates for species *k*, model (3) results in a U-shaped response function iff ${\hat{\beta }}_{2k}>0$.

The rationale of the use of the LLR criterion Eq (1) can be understood as follows. A large LLR indicates that it is advantageous to model each species with a separate model (*p*_*k*_). Hence, maximizing LLR(*α*) as a function of *α* will result in the environmental gradient that maximally separates the species-specific models. By formulating the criterion in terms of general distribution functions *p*_*k*_, a very flexible method arises. Here we restrict the *p*_*k*_’s to be Poisson distributions, but e.g. Zhang and Thas [17] studied the use of the zero-inflated Poisson (ZIP) distribution and Zhu et al. [15] even replaced *p*_*k*_ with nonparametric estimates. In this paper, however, the method is confined to the Poisson distribution and the Gaussian response function so that our method can be easily interpreted as an extension of the traditional ordination methods. The formulation of the constrained ordination method in terms of likelihoods allows extending the method by making use of the rich theory of likelihood-based methods. In this paper, we suggest to estimate the *β*_2*k*_ parameters of model (3) with a penalised maximum likelihood method that favors negative parameter estimates and hence results in bell-shaped response functions. Details are given in the next section.

Although our methods are not restricted for use in the quadratic model (3), they will be demonstrated with model (3). In general we say that ***β***_*k*_ and **w**_*i*_ are *q*-dimensional.

### Penalised Maximum Likelihood

The penalised maximum likelihood estimation procedure is inspired by a Bayesian setup. Let *g*(***β***_*k*_) denote the prior distribution on ***β***_*k*_. Although we only require penalisation on *β*_2*k*_ we will present the method more generally so that penalisation on other *β* parameters becomes also possible. The penalised maximum likelihood estimator is then defined as ***β***_*k*_ that maximises the posterior *p*_*k*_(***β***_*k*_ ∣ *y*, *z*) ∝ *p*_*k*_(*y* ∣ *z*,***β***_*k*_)*g*(***β***_*k*_).

Penalised Poisson regression has been described before in the literature [18, 19], but the focus is often on the L1 (lasso) and L2 (ridge) penalties that result from a Laplace and a normal prior, respectively. These priors have zero means which leads to estimates that are shrunken towards zero. In the present setting, however, we want to favour negative parameter values. Therefore we opt for a normal distribution with a negative mean.

We will describe two algorithms. The first algorithm relies on standard software for the estimation of the *β* parameters, whereas the second algorithm is an alternative formulation of the iterative reweighted least squares procedure that often forms the core of the estimation algorithm in statistical software.

Algorithm 1

Consider the score equation for parameter *β*_*jk*_,

$$\begin{array}{c} \sum_{i=1}^{n}\frac{\partial \log p_{k}\left(\beta_{k}|y_{ik},z_{i}\right)}{\partial \beta_{jk}}=\sum_{i=1}^{n}\left(y_{ik}-\exp\left(\beta_{k}^{t}w_{i}\right)\right)w_{ij}+n\frac{\partial \log g(\beta_{k})}{\partial \beta_{jk}}=0. \end{array}$$ (4)

We propose the following algorithm:

1. estimate ***β***_*k*_ from the non-penalised regression. Denote this estimate by ${\tilde{\beta }}_{k}$;
2. let ${\tilde{\omega }}_{ik}=-n\frac{\partial \log g({\tilde{\beta }}_{k})}{\partial \beta_{jk}}/w_{ij}$ and set ${\tilde{y}}_{ik}=y_{ik}-{\tilde{\omega }}_{ik}$;
3. find ${\hat{\beta }}_{jk}$ from solving the score equation $\sum_{i=1}^{n}\left({\tilde{y}}_{ik}-\exp\left(\beta_{k}^{t}w_{i}\right)\right)w_{ij}=0$;
4. set ${\tilde{\beta }}_{k}={\hat{\beta }}_{k}$;
5. iterate through steps 2–4 until convergence.

When the prior *g* is a normal distribution with mean ***δ***^*t*^ = (*δ*_0_, *δ*_1_, *δ*_2_) and covariance matrix *γ*^−1^ ***D*** with *γ* a tuning parameter and ***D*** a diagonal matrix with elements *d*_0_, *d*_1_, *d*_2_, we find ${\tilde{\omega }}_{k}=\gamma \sum_{j=0}^{2}\left(\beta_{jk}-\delta_{j}\right)/\left(w_{ij}d_{j}^{2}\right)$. Since we only require penalisation for *β*_2*k*_ we set *d*_0_ = *d*_1_ = +∞ (in the limit), resulting in ${\tilde{\omega }}_{k}=\gamma \left(\beta_{2k}-\delta_{2}\right)/\left(w_{i2}d_{2}^{2}\right)$. In this case $\gamma /d_{2}^{2}$ may be replaced by a single penalisation parameter, e.g. by setting *d*_2_ = 1. The prior parameter *δ*_2_ must be negative to favour negative ${\hat{\beta }}_{2k}$’s.

Algorithm 2

Eq (4) may be written as (for all *β*_*jk*_, *j* = 1, …, *q*, simultaneously)

$$\begin{array}{c} \sum_{i=1}^{n}\frac{\partial \log p_{k}\left(\beta_{k}|y_{ik},z_{i}\right)}{\partial \beta_{k}}=\sum_{i=1}^{n}\left(y_{ik}-\exp\left(\beta_{k}^{t}w_{i}\right)\right)w_{i}-n\gamma D^{-1}\left(\beta_{k}-\delta \right)=0. \end{array}$$ (5)

Upon applying a first order Taylor expansion of $\exp(\beta_{k}^{t}w_{i})$ about a fixed ***β***_*k*_, say ${\tilde{\beta }}_{k}$, the estimation equation becomes approximately

$$\begin{array}{c} \sum_{i=1}^{n}\frac{\partial \log p_{k}\left(\beta_{k}|y_{ik},z_{i}\right)}{\partial \beta_{k}}={\left(Y-{\tilde{D}}_{1k}{\tilde{\lambda }}_{k}\right)}^{t}W-\beta_{k}^{t}\left(W^{t}{\tilde{D}}_{2k}W+n\gamma D^{-1}\right)+n\gamma \delta^{t}D^{-1}=0, \end{array}$$ (6)

where ***W*** is the *n* × *q* matrix with rows $w_{i}^{t}$ (*i* = 1, …, *n*), and

$$\begin{array}{c} {\tilde{\lambda }}_{k}=\exp\left(W{\tilde{\beta }}_{k}\right)\;\;\;{\tilde{D}}_{1k}=\text{Diag}\left(1-{\tilde{\beta }}_{k}^{t}w_{i}\right)\;\;\;{\tilde{D}}_{2k}=\text{Diag}\left(\exp\left({\tilde{\beta }}_{k}^{t}w_{i}\right)\right). \end{array}$$ (7)

A proof is provided in S1 Text.

Given a ${\tilde{\beta }}_{k}$ for the calculation of ${\tilde{\lambda }}_{k}$, ${\tilde{D}}_{1k}$ and ${\tilde{D}}_{2k}$, Eq (6) results in

$$\begin{array}{c} {\hat{\beta }}_{k}={\left(W^{t}{\tilde{D}}_{2k}W+n\gamma D^{-1}\right)}^{-1}\left[{\left(Y-{\tilde{D}}_{1k}{\tilde{\lambda }}_{k}\right)}^{t}W+n\gamma \delta^{t}D^{-1}\right]. \end{array}$$ (8)

The ***β***_*k*_ parameters can thus be estimated by iteratively calculating Eqs (7) and (8); the algorithm can be initialised by choosing ${\tilde{\beta }}_{k}$ to be the not-penalised MLE.

### Estimation of the Environmental Gradient

The LLR criterion Eq (1) is now defined in terms of the posteriors,

$$\begin{array}{c} \text{LLR}\left(\alpha \right)=\sum_{i=1}^{n}\sum_{k=1}^{s}\left(\log p_{k}\left(y_{ik}|z_{i},\beta_{k}\right)+\log g\left(\beta_{k}\right)-\log p\left(y_{ik}|z_{i},\beta \right)\right). \end{array}$$ (9)

The LLR has to be maximised in terms of ***α***, but note that the terms originating from the priors do not depend on ***α***. Hence, Eq (9) reduces to Eq (1).

In S2 Text we give details on a convenient iterative estimation algorithm based on Fisher scoring.

Often more than one environmental gradient is required to understand the species-environment relationship. We propose to work along the lines of Zhu et al. [15] and ter Braak and Prentice [20]. The coefficients of the first environmental gradient, say ***α***_1_ is obtained by maximising the LLR criterion Eq (9) and it results in the environmental scores $z_{1i}=\alpha_{1}^{t}x_{i}$ (*i* = 1, …, *n*).

The second environmental score must provide new information, unrelated to the first dimension. To this end, the environmental variables ***x***_*i*_ are regressed on the scores of the first dimension. In particular, the *p* regression models, *x*_*ij*_ = *ζ*_0*j*_ + *ζ*_1*j*_ *z*_1*i*_+*ϵ*_*ij*_ (*j* = 1, …, *p*), with E{*ϵ*_*ij*_} = 0 are fitted using ordinary least squares. The resulting residuals, $e_{ij}=x_{ij}-{\hat{\zeta }}_{0j}-{\hat{\zeta }}_{1j}z_{1i}$, are known to be uncorrelated with the regressor (environmental score) of the first dimension. The matrix ***X*** is now replaced with the matrix ***E*** with rows $e_{i}^{t}=\left(e_{i1},\ldots ,e_{ip}\right)$ and this matrix serves as the new environmental matrix for obtaining the second environmental gradient. More gradients can be found by repeating this procedure (regressing ***x*** on all environmental scores).

## Results

### Simulation Study

#### Simulation Setup

In this simulation study we evaluate the new method empirically.

Data will be simulated for species with bell-shaped response functions and for species with U-shaped response functions. Parameters are chosen such that the U-shaped functions can be better separated than the bell-shaped functions, along some gradient. Classical methods are thus expected to find this gradient, whereas the BECOA method is designed to find another gradient along which mostly the bell-shaped functions are well separated.

We proceed with the following steps for the generation of the simulated data: (1) construct an environmental data matrix, ***X***, with observations of *p* = 4 environmental variables measured on *n* = 44 sampling locations; (2) specify two environmental gradients (***α***_1_ and ***α***_2_); (3) specify *s* = 20 bell-shaped and U-shaped species response functions along the environmental gradients; (4) simulate 44 abundances for each of the 20 species. Details follow.

**Fig 2:**
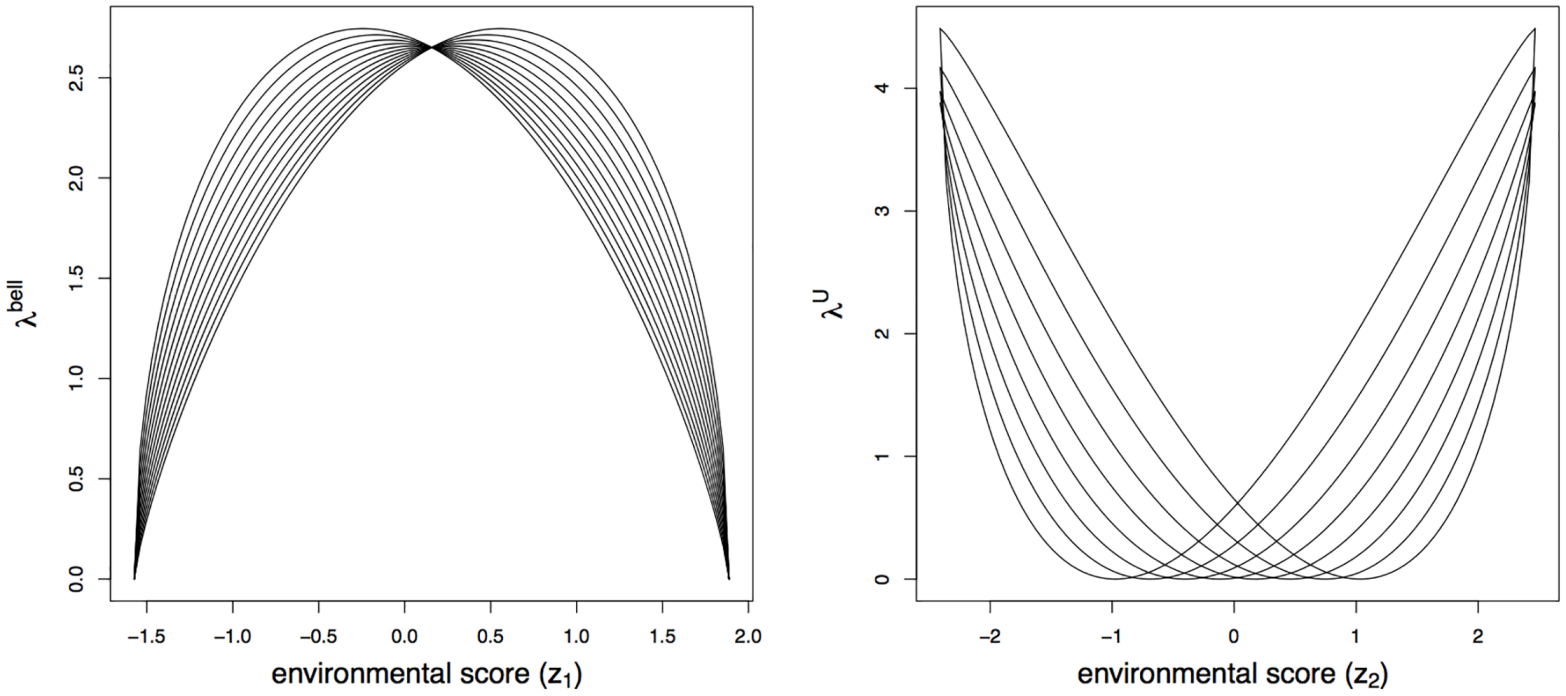
The bell-shaped response functions of the first twelve species (left panel) and the U-shaped response functions of the next eight species (right panel).

1. The 44 × 4 matrix ***X*** is formed by the four principle components of a part of the environmental data matrix used in Section. The measurements of calcium (Ca^2+^), magnesium (Mg^2+^) and potassium (K^+^) and Silicate were considered.
2. The environmental gradients are orthonormal and set to $\alpha_{1}^{t}=\left(1/\sqrt{2},-1/\sqrt{2},0,0\right)$ and $\alpha_{2}^{t}=\left(0,0,1/\sqrt{2},-1/\sqrt{2}\right)$. The 44 corresponding environmental scores on the first two dimensions are calculated as ***z***_1_ = ***X*** ***α***_1_ and ***z***_2_ = ***X*** ***α***_2_.
3. The response functions of the first twelve species are bell-shaped *β*-functions. In particular, for species *k* = 1, …, 12,
  $$\begin{array}{c} \text{E}\left\{Y|z\right\}=f_{k}^{\text{bell}}\left(z\right)=s_{k}{\left(z-a\right)}^{\eta_{k}}{\left(b-z\right)}^{\zeta_{k}}\;a\leq z\leq b, \end{array}$$ (10)
  where for scale parameter *s*_*k*_ > 0 the expected abundance is always positive and it reaches its minimal value 0 at the two boundaries *a* and *b*. The function reaches its maximum at $z=\frac{a\zeta_{k}+b\eta_{k}}{\eta_{k}+\zeta_{k}}$ (optimum); this maximum equals $\frac{{\left(\eta_{k}\left(b-a\right)\right)}^{\eta_{k}}{\left(\zeta_{k}\left(b-a\right)\right)}^{\zeta_{k}}}{{\left(\eta_{k}+\zeta_{k}\right)}^{\eta_{k}+\zeta_{k}}}$. Table 1 shows the parameter settings for the first twelve species.
  The remaining eight species show U-shaped response functions. In particular, for species *k* = 13, …, 20,
  $$\begin{array}{c} \text{E}\left\{Y|z\right\}=f_{k}^{\text{U}}\left(z\right)=s_{k}\left(c-{\left(z-a\right)}^{\eta }{\left(b-z\right)}^{\zeta_{k}}\right)\;a\leq z\leq b, \end{array}$$ (11)
  where $c=\frac{{\left(\eta_{k}\left(b-a\right)\right)}^{\eta_{k}}{\left(\zeta_{k}\left(b-a\right)\right)}^{\zeta_{k}}}{{\left(\eta_{k}+\zeta_{k}\right)}^{\eta_{k}+\beta_{k}}}$ is a location parameter to ensure positive abundances for all *z* ∈ [0, 1]. Table 2 shows the parameter settings for the eight species with U-shaped response functions.
4. The abundances of the first twelve species are randomly generated from a Poisson distribution with mean parameter set to $f_{k}^{\text{bell}}\left(z_{1i}\right)$ with *z*_1*i*_ the environmental scores along the first gradient (*k* = 1, …, 12). The abundances of the other eight species use mean parameter $f_{k}^{\text{U}}\left(z_{2i}\right)$ with *z*_2*i*_ the environmental scores along the second gradient (*k* = 13, …, 20).
  The response functions are depicted in Fig 2. The parameters were chosen so that the U-shaped response functions are more separated than the bell-shaped response functions. Hence, the most important gradient found by the FCOA is more likely to be the second gradient, whereas the new method is expected to detect the first gradient.

**Table 1:** The parameters used for the bell-shaped response functions for species *k* = 1, …, 12. For all species, the scaling parameters *s*_*k*_ are set to (*η*_*k*_+*ζ*_*k*_) so as to make the maxima comparable.

	Species *k*											
	1	2	3	4	5	6	7	8	9	10	11	12
*η*_*k*_	0.50	0.53	0.55	0.58	0.61	0.64	0.66	0.69	0.72	0.75	0.77	0.80
*ζ*_*k*_	0.80	0.77	0.75	0.72	0.69	0.66	0.64	0.61	0.58	0.55	0.53	0.50
*s*_*k*_	1.30	1.30	1.30	1.30	1.30	1.30	1.30	1.30	1.30	1.30	1.30	1.30
optimum	-0.24	-0.17	-0.10	-0.02	0.05	0.12	0.19	0.27	0.34	0.41	0.48	0.56

**Table 2:** The parameters used for the U-shaped response functions for species *k* = 13, …, 20. For all species, the scaling parameters *s*_*k*_ are set to $\frac{\eta_{k}+\zeta_{k}}{2}$ so as to make the maxima comparable.

	Species *k*							
	13	14	15	16	17	18	19	20
*η*_*k*_	0.50	0.60	0.70	0.80	0.90	1.00	1.10	1.20
*ζ*_*k*_	1.20	1.10	1.00	0.90	0.80	0.70	0.60	0.50
*s*_*k*_	0.85	0.85	0.85	0.85	0.85	0.85	0.85	0.85
optimum	-0.98	-0.69	-0.40	-0.11	0.17	0.46	0.75	1.03

Thousand datasets have been generated according to this procedure, and for each dataset the CCA, FCOA, and the new BECOA methods have been applied. Only the first environmental gradient is evaluated in the simulation study. Later, in the example section the use of the second dimension will be illustrated.

#### Results

**Fig 3:**
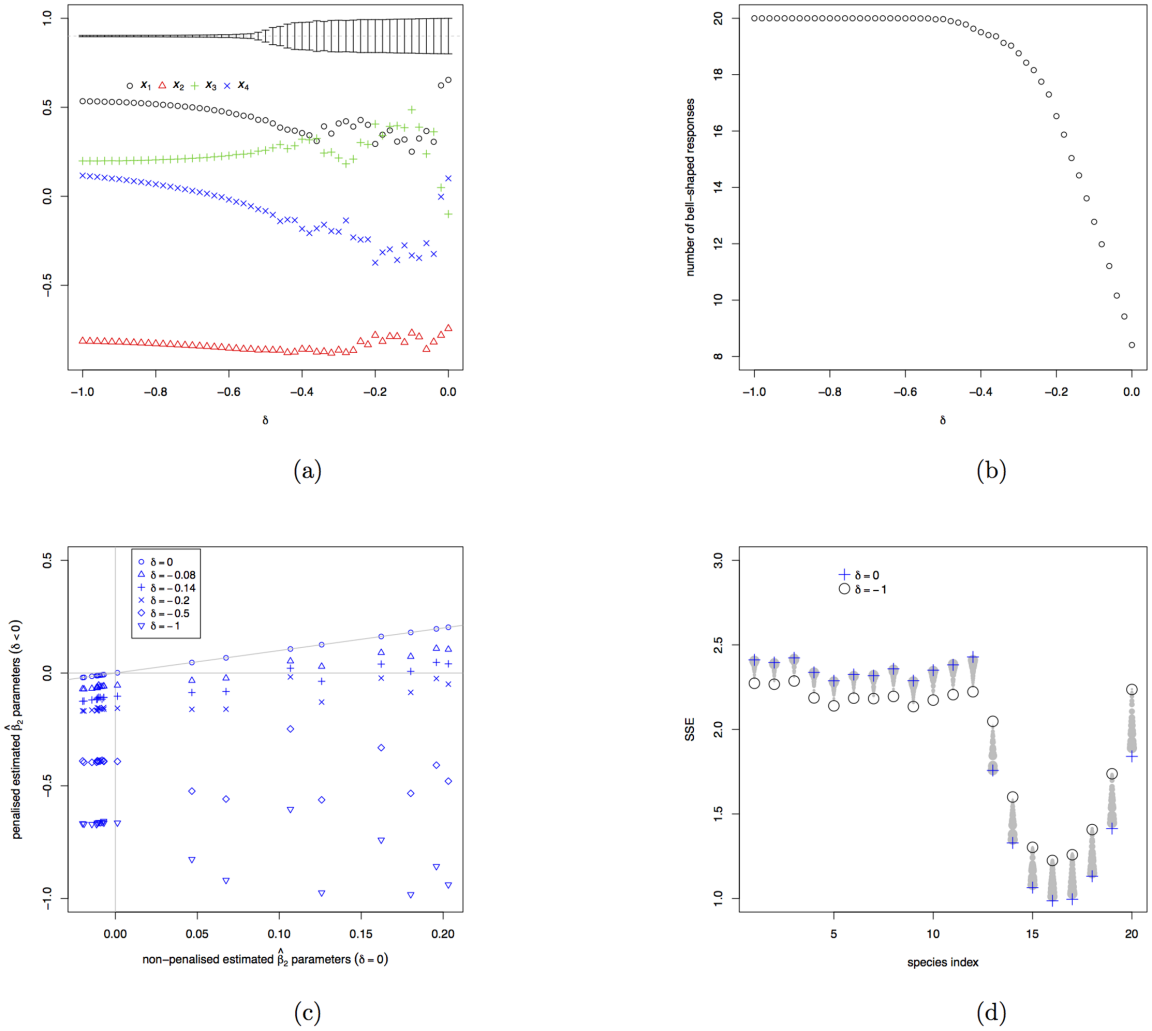
Results of the simulation study. (a) the averages of the estimated environmental gradients as a function of the penalty parameter *δ*; the intervals shown on top are proportional to the total variance of the estimates. (b) the average number of bell-shaped response functions as a function of the penalty parameter *δ*. (c) for each of the 20 species the graph shows the evolution of the ${\hat{\beta }}_{2k}$’s as *δ* changes. (d) for each of the 20 species the graph shows the evolution of the Sum of Squared Errors (SSE) of the fits of the response functions for the penalty parameter moving from *δ* = 0 (symbol: +) to *δ* = −1 (symbol: O); the dots represent the intermediate results with larger dots representing smaller penalisation.

For each of the thousand generated datasets, the penalization parameter *δ* is set to vary from 0 to −1 in steps of −0.02. Fig 3(a) shows how the penalty affects the coefficients as estimated by the new BECOA method. As the penalization becomes stronger (i.e. moving from *δ* = 0 to larger negative *δ*’s), the estimates start deviating until they become stable for *δ* < −0.6. The nervous behaviour of the coefficient estimates for −0.6 < *δ* < 0 agrees with the large variance of these estimates (see intervals at the top of Fig 3(a)).

Fig 3(b) demonstrates that the new estimation method succeeds in increasing the number of species with bell-shaped response functions. Without penalisation only about eight species have bell-shaped response functions on average, but for *δ* < −0.5 all 20 species do so in almost all simulations. Fig 3(c) shows how the ${\hat{\beta }}_{2k}$ are affected by the penalisation. Having forced all species to have bell-shaped response functions is not necessarily good. Therefore we have also assessed the goodness-of-fit with the total sum of squared errors (SSE) calculated from the fits of all 20 response functions: the effect of the penalisation on the goodness of fit is illustrated in Fig 3(d). It shows that the fit of the response functions of species 1–12 (with genuine bell-shaped response functions) slightly improves with increasing penalty, whereas for the other eight species the penalisation has a negative effect.

**Fig 4:**
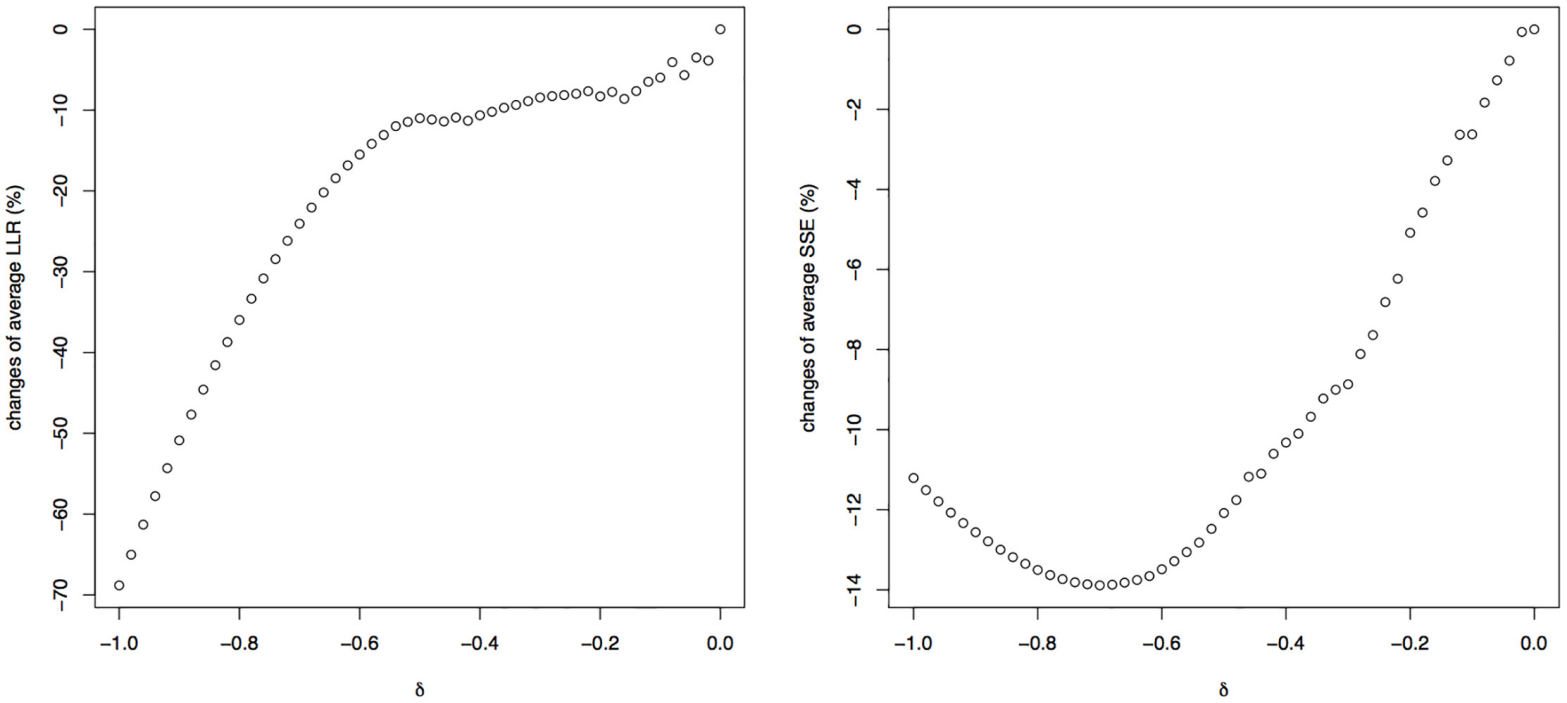
The relative changes of average LLR (left) and average SSE (right) as a function of the penalty parameter *δ*.

Fig 4 provides two diagnostic graphs that may be used for choosing an appropriate value for the penalisation parameter *δ*. The goal of our method is to find the gradient along which the species abundance distributions are maximally separated as measured by the LLR. However, only the contributions made by species with bell-shaped response functions should be included, because no ecologically meaningful interpretation will be given to the other species. We therefore define the average LLR (aLLR) as the LLR of Eq (9), but excluding the species with *U*-shaped fitted response functions, and divided by the number of bell-shaped fitted response functions. The left panel of Fig 4 shows a graph of the relative change of aLLR plotted against the penalty parameter. The relative change is computed as $\frac{\text{average}\;\text{LLR}(\delta )-\text{average}\;\text{LLR}(\delta =0)}{\text{average}\;\text{LLR}(\delta =0)}$. Hence, a minus sign in the percent change indicates the separation of the bell-shaped response functions at *δ* is worse than at *δ* = 0. The construction of the right panel of Fig 4 is similar, but showing the average SSE (aSSE) as a measure for the quality of fit of the bell-shaped response functions. For this simulation study, the left panel of Fig 4 suggests that for *δ* > −0.6 the penalisation has hardly a negative effect on the separation of the bell-shaped response functions, whereas the right panel indicates that the penalisation has a positive effect on the quality of the fit for species with bell-shaped response functions.

We conclude that, for the settings of the simulation study, the method works well. For *δ* = −0.6, almost all species can have bell-shaped response functions, with overall a good goodness-of-fit, while loosing only about 10% of separation between the response functions.

### Example

We consider data from a limnology study conducted by Verleyen et al. [21]. They collected 45 water samples from lakes in ice-free regions along the east Antarctic coastline. Within the water samples seven physical and chemical characteristic were measured (environmental variables), including the concentrations of major ions (NH_4_^+^, K^+^, Mg^2+^, Ca^2+^ and Cl^-^), silicate and the dissolved organic carbon (DOC). More than 500 microbial species were identified using a Massive Parallel Sequencing technique (Roche 454) and their relative abundances were calculated. Here we only use data from the species with more than three nonzero records, resulting in a reduction to 199 species. Each of the environmental variables was standardised to zero mean and unit variance prior to analysis. The data are analysed with three methods: BECOA, CCA and FCOA. For the latter method we considered a quadratic log-linear Poisson regression model as in Eq (3). All calculations are performed with R [22]; for CCA the *vegan* R package has been used.

#### Selection of the tuning parameter

**Fig 5:**
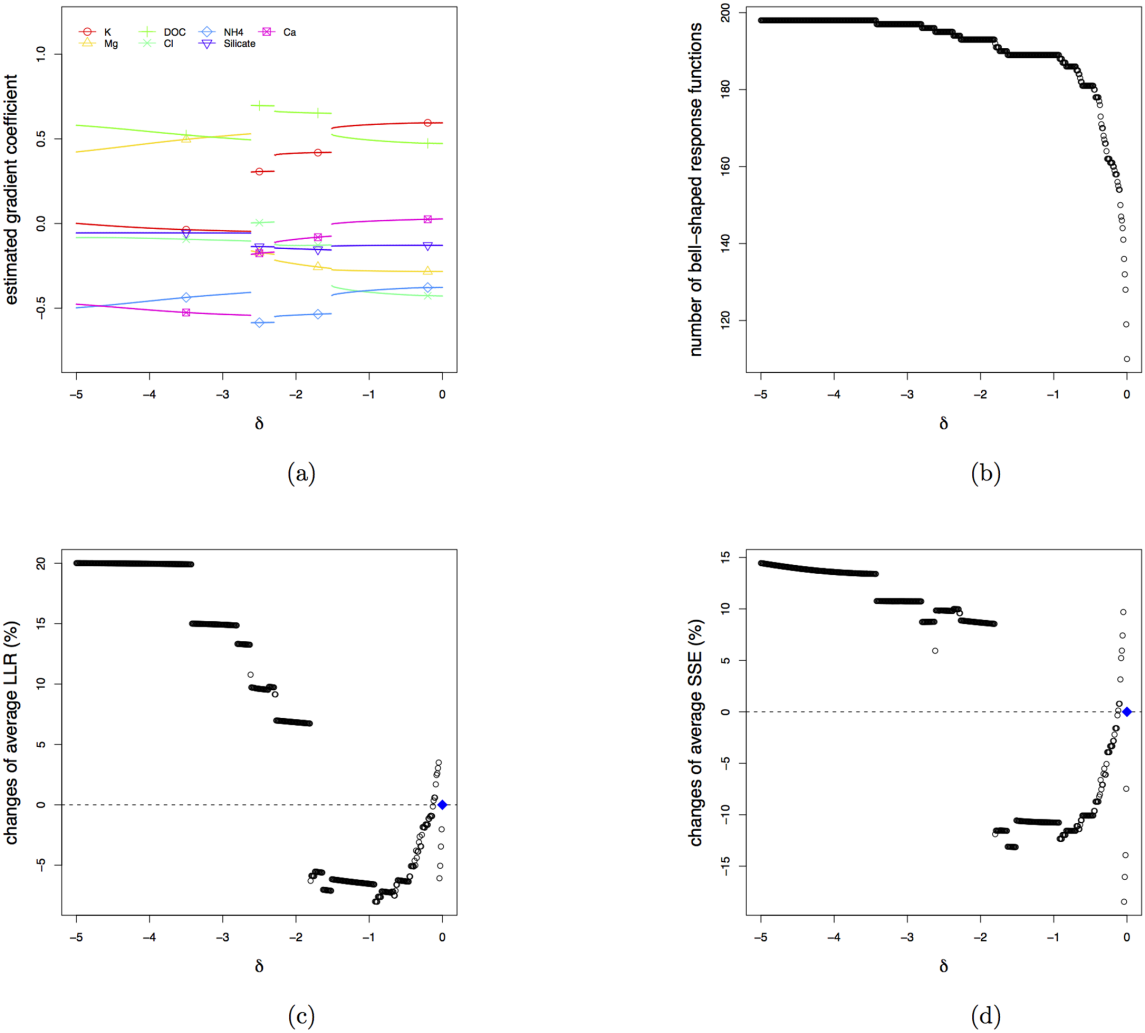
Results for the case study in the first dimension. Estimated coefficients of environmental gradient (a) and the average number of bell-shaped response functions (b) as a function of penalty parameter *δ*. Relative changes of average LLR (c) and average SSE (d) as a function of penalty parameter *δ*.

The normalised estimated coefficient of the environmental gradient for the first dimension are presented in Fig 5(a) as a function of the *δ* penalty parameter. All coefficients are affected by *δ* and the adjustment is sometimes quite substantial (e.g. the sign of coefficient of Mg^2+^ changed from negative to positive). Fig 5(b) demonstrates a large increase in the number of species with bell-shaped response functions as the penalisation becomes heavier. At about *δ* = −3.5 this number reaches 199. From Fig 5(c) we see that the average LLR climbs with increasing penalisation, i.e. harder penalisation leads to more discrimination between the species with bell-shaped response functions. Fig 5(d) shows the effect of penalisation on the average SSE. From this graph we conclude that, among the species with bell-shaped response functions, the aSSE quickly increases with penalisation, then (−2 < *δ* < −0.2) it decreases, before (*δ* < −2) increasing again.

**Fig 6:**
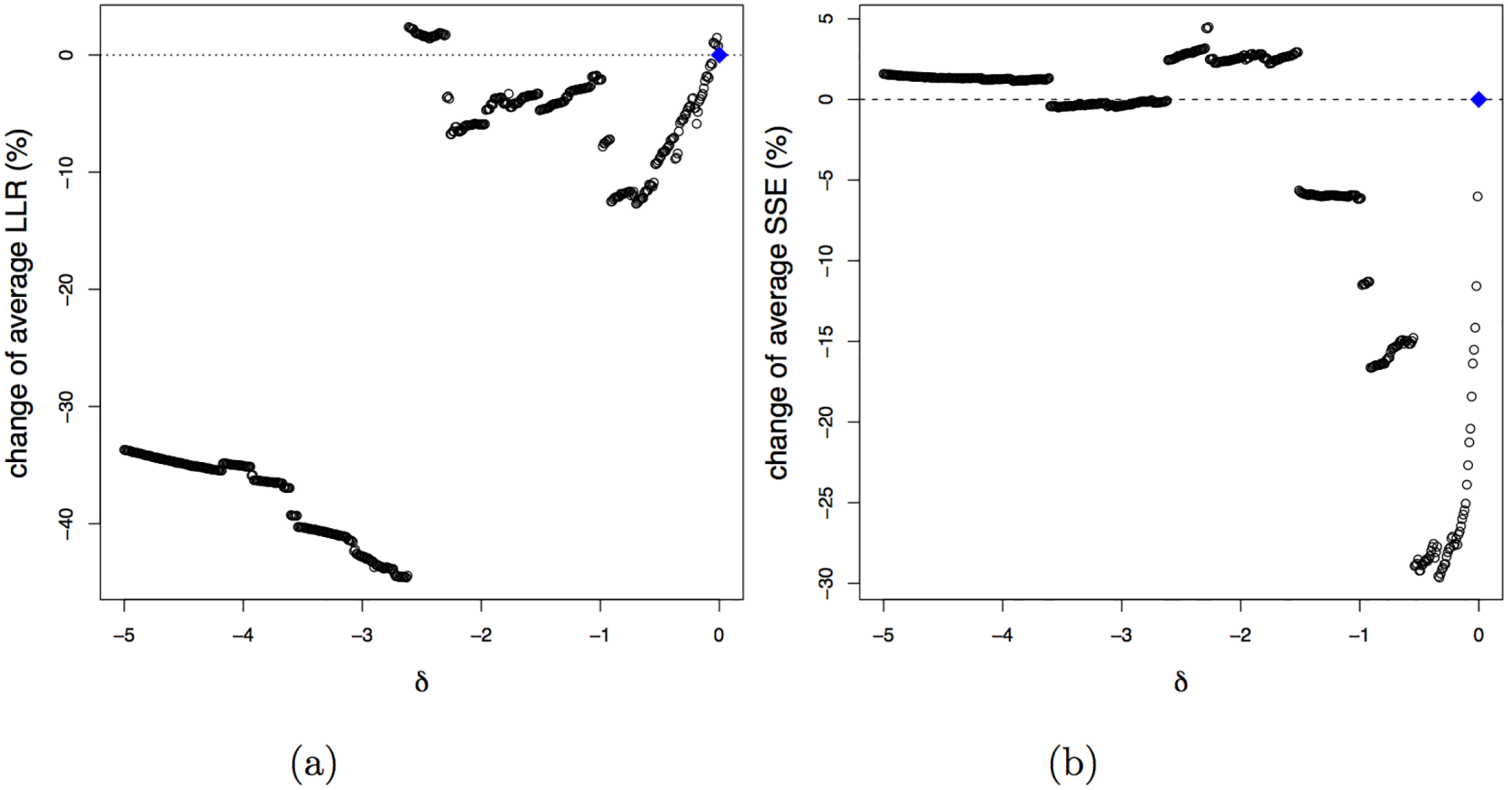
Cross-validated (10-fold) results for the case study in the first dimension. Relative change of the average LLR (a) and average SSE (b) as a function of the penalty parameter *δ*.

Fig 6 shows the aLLR and aSSE, but now computed by 10-fold cross-validation so as to give a more honest assessment. See S3 Text for details. From this graph we conclude that a compromise may be obtained with *δ* ∈ [−2.5; −1], resulting in an increase in the number of bell-shaped response functions from 110 to 190 with minimal cost in terms of quality of fit and discrimination.

**Fig 7:**
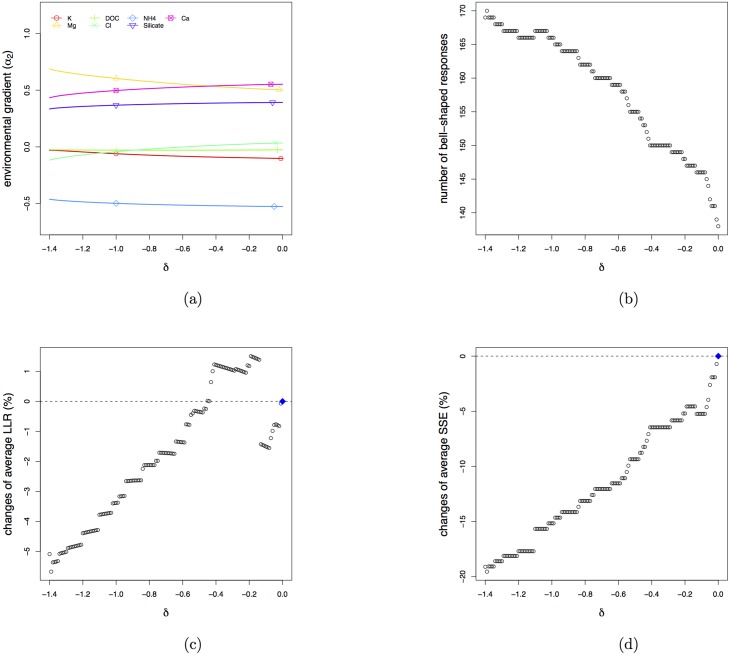
Results for the case study in the second dimension. Estimated coefficients of environmental gradient (a) and the average number of bell-shaped response functions (b) as a function of penalty parameter *δ*. Relative changes of average LLR (c) and average SSE (d) as a function of penalty parameter *δ*.

Fig 7 shows the result for the second dimension. Fig 7(a) reveals that additional to the important environmental variables displayed in Fig 5(a), Silicate plays an important role in the second most discriminating direction. Along the second gradient we can obtain at most 170 species with bell-shaped response functions (Fig 7(b)) at the cost of about 5% reduction in the aLLR (Fig 7(c)). The aSSE, as shown in Fig 7(d), falls down to 20% in almost a linear way.

**Fig 8:**
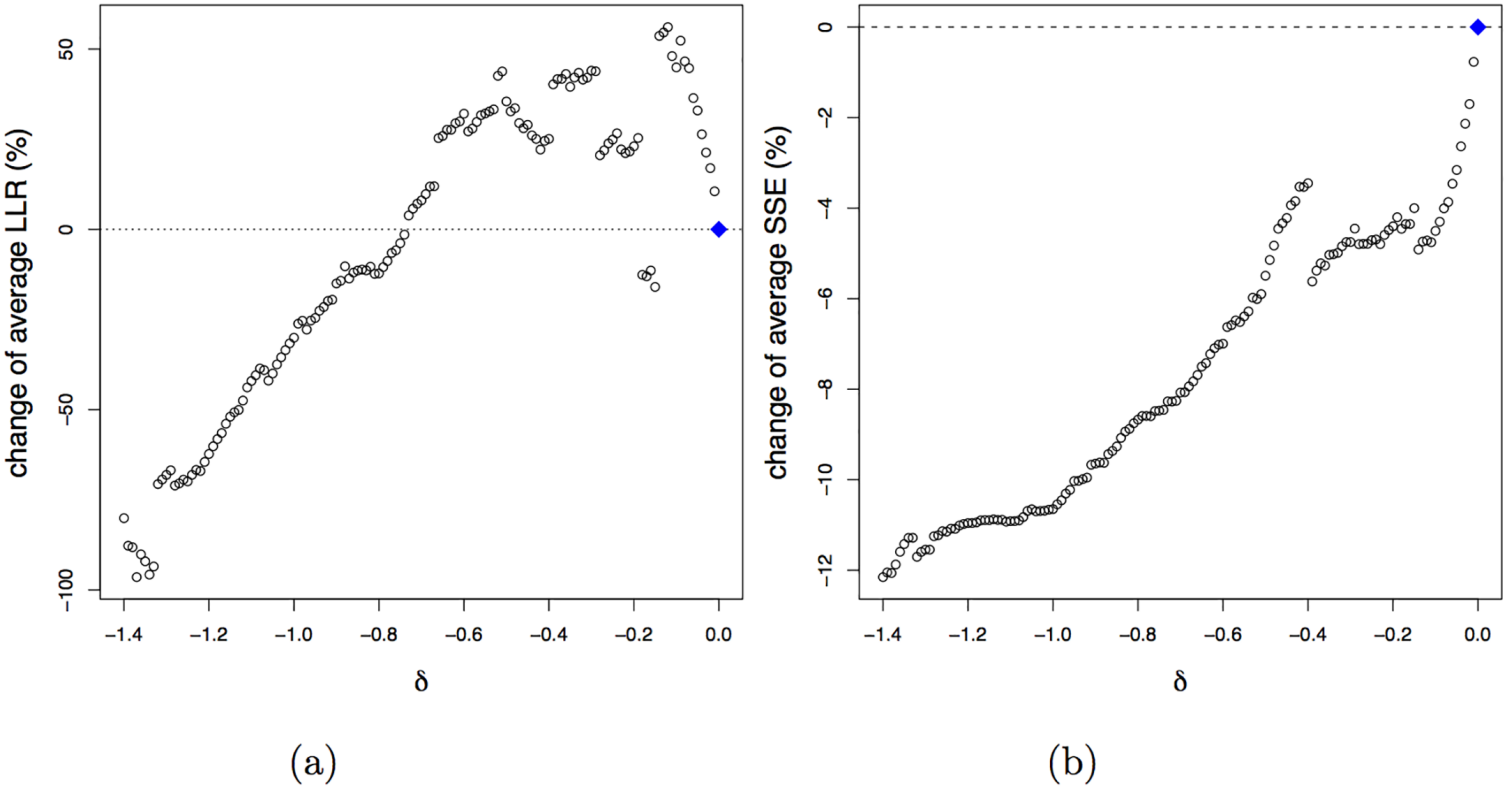
Cross-validated (10-fold) results for the second dimension. Relative change of the average LLR (a) and average SSE (b) as a function of the penalty parameter *δ*.

The cross-validated aLLR and aSSE (Fig 8) display similar descending trends. This brings us to an appropriate *δ* of about −0.7, resulting in no cost in terms of separation of the bell-shaped response functions and an increase of aSSE of about 10%, while more than 20 additional species are modelled with bell-shaped response functions.

#### Discussion

The estimated coefficients from three different ordination methods are listed in Table 3. The new method clearly has triggered changes in the estimates. Based on the discussion from the previous section, we have selected *δ* = −1.7 and *δ* = −0.7 for dimensions 1 and 2, respectively.

**Table 3:** Comparison of the estimated environmental gradients and the model fits from three ordination methods applied to the Antarctic lakes data. Dimension 1 and Dimension 2 refer to models fitted with the environmental scores on dimensions 1 and 2, respectively. MSE gives the mean squared error calculated only among Bell-shaped species, MSE* stands for the mean squared error calculated from all species.

	Dimension 1			Dimension 2		
	BECOA	FCOA	CCA	BECOA	FCOA	CCA
number	190	80	199	166	66	199
$\sqrt{\text{MSE}}$	127.57	181.76	161.88	129.76	168.75	161.71
$\sqrt{{\text{MSE}}^{*}}$	156.38	143.94	161.88	152.73	157.16	161.71
Estimated environmental gradient						
	Dimension 1			Dimension 2		
	BECOA	FCOA	CCA	BECOA	FCOA	CCA
K^+^	0.4186	0.4409	0.1939	-0.0783	0.6754	0.2814
Mg^2+^	-0.2565	-0.1866	-0.0275	0.5640	0.1272	-0.0399
DOC	0.6525	0.6056	-0.3890	-0.0291	-0.2000	-0.5647
Cl^-^	-0.1280	-0.3797	-0.0181	-0.0098	0.5804	-0.0262
NH_4_^+^	-0.5355	-0.4861	0.4581	-0.5108	-0.1743	0.6650
Silicate	-0.1536	-0.1534	-0.0236	0.3784	0.2051	-0.0343
Ca^2+^	-0.0808	-0.0073	-0.2723	0.5209	-0.2799	-0.3953

For the first environmental gradient direction we conclude that FCOA is not too dissimilar from the new method, whereas CCA gives quite different results. Table 3 also gives a closer insight into the consequences of the differences. The apparently small difference between BECOA and FCOA corresponds to a more than doubling of the number of bell-shaped response functions, in favour of BECOA. Moreover, among the bell-shaped response functions, the quality of the fit of those from BECOA is better. By construction CCA fits bell-shaped response functions to all species, but the average MSE is worse than with BECOA.

Along the second dimension all three methods give different solutions. Table 3 shows that BECOA succeeds in fitting 166 bell-shaped response functions, whereas FCOA has only 66. As for the first dimension, BECOA gives the smallest average MSE among the bell-shaped response functions.

In S1 Table results of the joint model fits for the first and the second dimension are given for all three ordination method. Again BECOA appears on top in terms of MSE among the bell-shaped response functions, while FCOA has the poorest performance.

#### Ordination diagram

**Fig 9:**
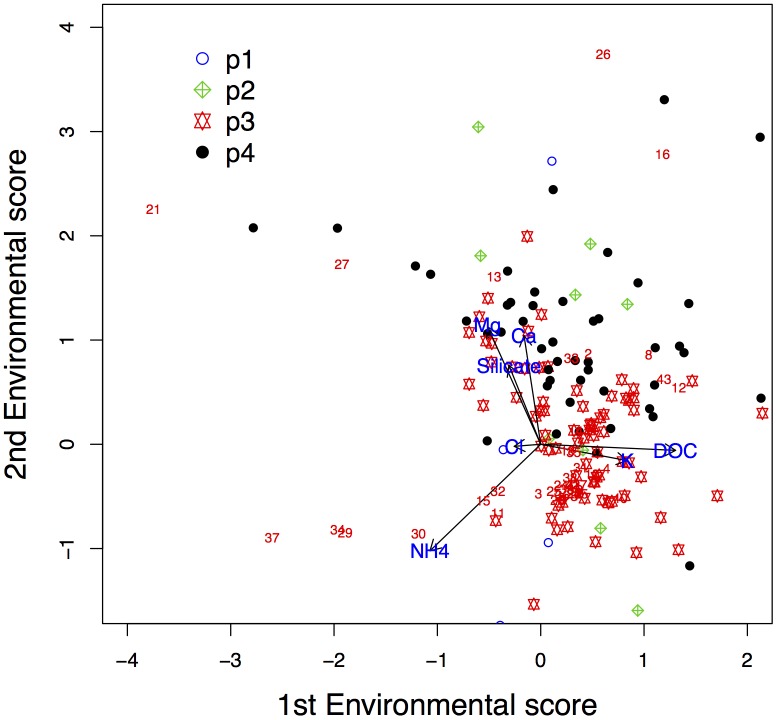
Ordination diagram of the BECOA analysis of the Antarctic lake data, with penalisation parameter *δ* being -1.7 for the first dimension and = 0.7 for the second dimension. Numbers represent lakes. The points represent the species optima, with symbols indicating the shape of the corresponding species response function when *δ* = 0: p1, U-shaped in 1st and 2nd dimension; p2, bell-shaped in 1st dimension; p3, bell-shaped in 2nd dimension; p4, bell-shaped in 1st and 2nd dimension.

The results from the previous section are graphically presented as an ordination graph, similar as in Yee [23]; see Fig 9. Information about the species optima, sampling sites and environmental gradients can be read from the graph. We argue that the data-analyst should only look at species for which a bell-shaped response function is obtained. Hence, only the 159 species for which in both ordination dimensions bell-shaped response functions were found, are plotted. The plotting symbols indicate in what dimensions their response functions were U or bell-shaped if no penalisation were applied (*δ* = 0).

**Fig 10:**
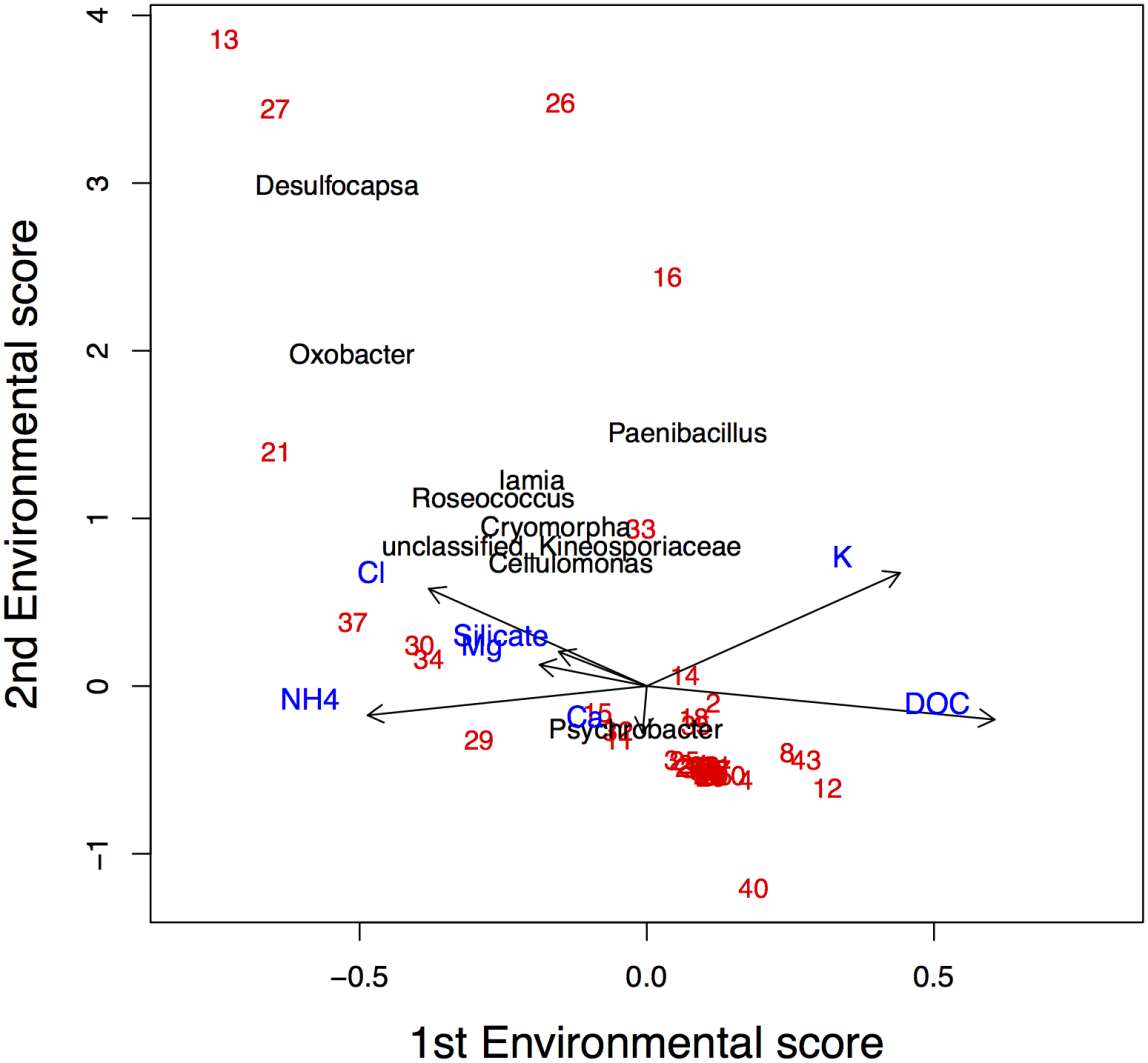
Ordination diagram of the CCA analysis of a subset of Antarctic data.

**Fig 11:**
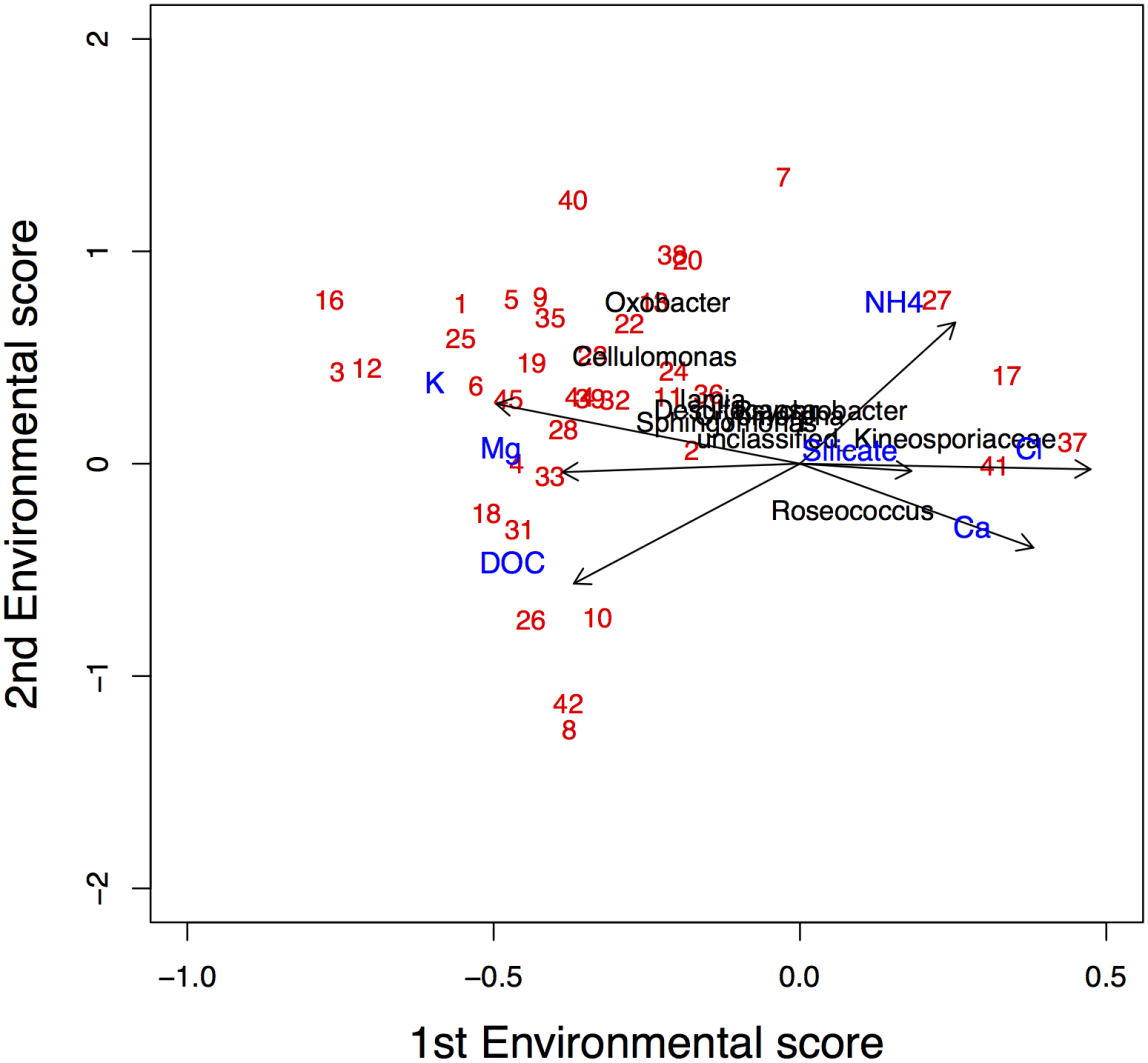
Ordination diagram of the FCOA analysis of a subset of Antarctic data.

This allows for deducing species-site relationships and the importance of environmental variables on species abundance distributions. The ordination diagrams of CCA and FCOA are included as Figs 10 and 11

**Fig 12:**
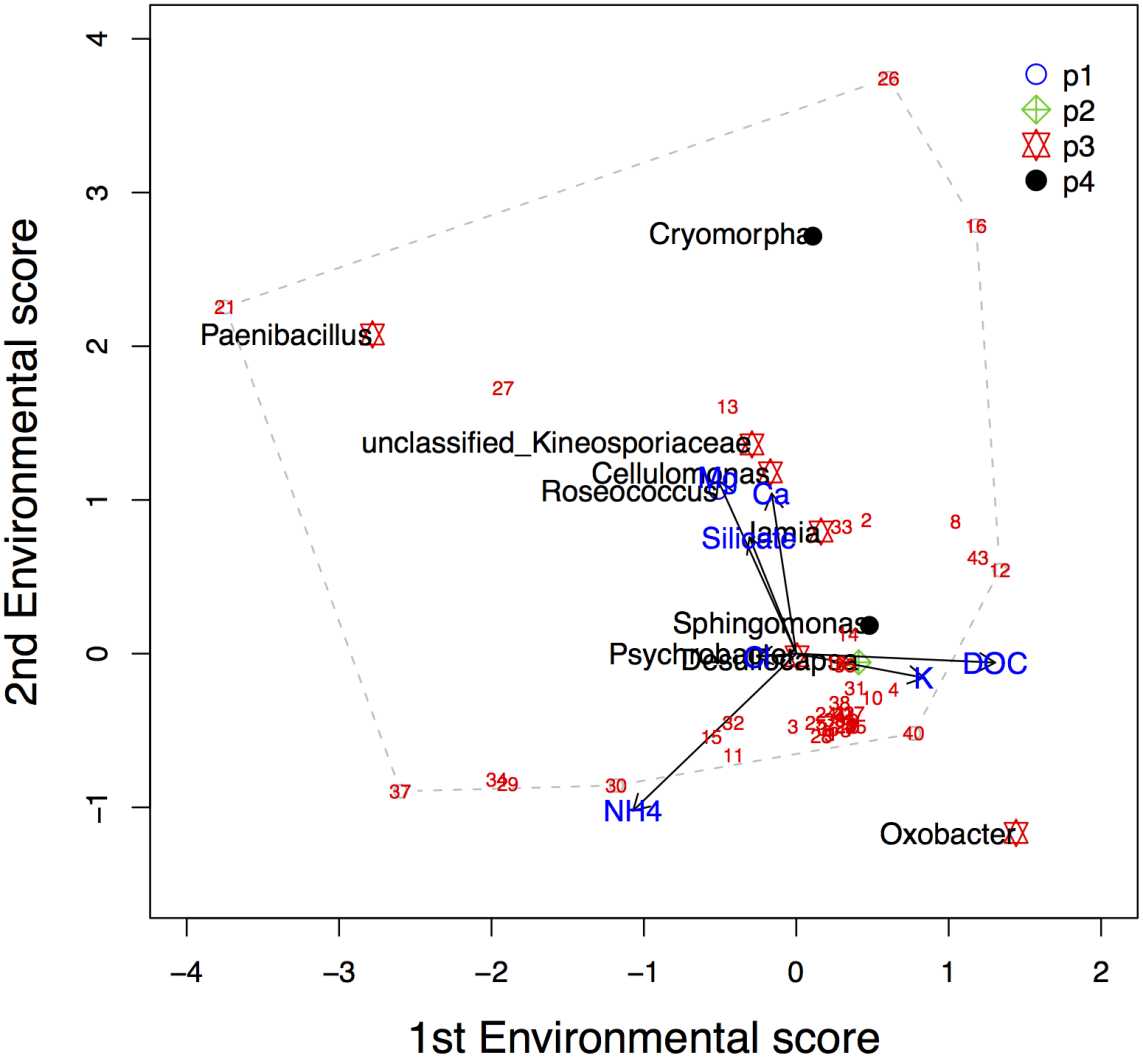
Ordination diagram of the BECOA analysis of a subset of the Antarctic lake data, with penalisation parameter *δ* being -1.7 for the first dimension and = 0.7 for the second dimension. Numbers represent lakes. The points represent the species optima, with symbols indicating the shape of the corresponding species response function when *δ* = 0: p1, U-shaped in 1st and 2nd dimension; p2, bell-shaped in 1st dimension; p3, bell-shaped in 2nd dimension; p4, bell-shaped in 1st and 2nd dimension. Species labels are added.

Due to the large number of species the readability of Fig 9 is poor. In order to increase the interpretability, an ordination diagram with only ten randomly-selected species is presented in Fig 12. We conclude that particularly NH_4_^+^, K^+^ and DOC determine the first dimension, while Mg^2+^, Ca^2+^ and NH_4_^+^ dominate the second dimension.

For species *Iamia*, for instance, the graph suggests that this species is most abundant at locations 33 and 2. This is confirmed by the observed abundances of this species: 105 and 74 at sites 33 and 2, respectively.

The results of the CCA analysis can also be depicted in an ordination graph. However, instead we summarise the differences between CCA and BECOA in a few statistics. We calculated the Euclidean distances between species optima and sites scores for the ordination diagrams produced by CCA and the new method. For each species we calculated the log-ratios of the distance from CCA over the distance from BECOA. A log-ratio close to zero indicates that both methods give about the same conclusion with respect to the preference of that species for that particular site. In the first dimension the median is 0.11 and the first and third quartiles are −0.94 and 1.36. Thus half of the species-location pairs have distance-ratio’s more extreme than 0.11 and 23. For the second dimension, the median is 0.78 and the quartiles are −0.10 and 1.65, or half of the species-location pairs have distance-ratio’s more extreme than 0.80 and 45.

## Conclusion and Discussion

Constrained ordination analysis (COA) methods aim at finding dimensions in the environmental space along which the species abundance response functions are maximally separated, allowing for explaining differences between the species environmental niches. Results from such analyses are typically graphically displayed as a biplot, which shows dots for species optimal environmental conditions. However, some COA methods do not enforce bell-shapes, but they do provide parameter estimates used for the construction of the biplots, which, consequently, are misleading and conclusions are prone to error. Other methods do enforce the bell-shape, but by doing so they may result in poor fits of the response functions.

In this paper we have proposed a COA that searches for environmental gradients along with *bell-shaped* response functions are well separated. This is accomplished by setting up a tailored penalised maximum likelihood method that penalises U-shaped response functions. As a result environmental gradients can be identified that especially separate the ecologically meaningful bell-shaped response functions. We advise to remove species from the biplot for which no bell-shaped response functions are found for the first two environmental gradients.

In particular, two algorithms were proposed. The first method gives a simple implementation allowing for the use of Poisson regression routines available in most software packages. The second procedure requires the implementation of a variant of iteratively reweighted least squares, but it gives faster estimation times. In S5 Text we demonstrate the implementation of BECOA in R.

In Appendix B of ter Braak [24] a computationally efficient algorithm is proposed to find a least square estimator subject to both nonnegative and sum constraints based on the lasso-path algorithm [25]. Here, we do not necessarily need the sum constraint because sparseness will not further improve the interpretability of the ordination analysis, but it may be worthwhile to further investigate the method for imposing nonnegative (or, equivalently, nonpositive) parameter estimates and study how this can be extended to a likelihood setting. Due to the use of the flexible LLR criterion, the BECOA can be extended to accommodate non-parametrically modelled response function following Zhu et al. [15] or Eilers [26]. The latter proposed using B-splines and an asymmetric penalty for enforcing unimodal response functions.

Through a simulation study and a case study from a metagenomics aquatic limnology study, we have demonstrated the added value of our methods. For choosing an appropriate value of the penalisation tuning parameter, we have proposed a few diagnostic graphs related to the quality of the fit of the response functions and to the extent of separation of the bell-shaped response functions.

Although we only focused on modelling the species abundances, our method can be easily adapted to absence/presence data. This can be accomplished by replacing the Poisson regression with logistic regression (details are given in S4 Text). Similar adaptations may be considered when overdispersion (negative binomial regression) or an excess of zero abundances (zero inflated Poisson or zero inflated negative binomial regression) is expected.

## Supporting Information

S1 Text Iterative Reweighted Least Squares. (PDF)

S1 Table Joint model fits. (PDF)

S1 Data Set The Antarctic lakes data set. (XLSX)

S2 Text Newton-Raphson for the Maximisation of LR. (PDF)

S3 Text Cross validation. (PDF)

S4 Text Algorithm of absence/presence data. (PDF)

S5 Text Implementation in R. (PDF)
