# Supplementary material for: Constrained Ordination Analysis with Enrichment of Bell-Shaped Response Functions
Source: PLoS One. 2016 Apr 21;11(4):e0154079. doi: 10.1371/journal.pone.0154079 (PMC4839756; doi:10.1371/journal.pone.0154079)
Supplement: S1 Text — (PDF) [file pone.0154079.s001.pdf]

Supporting Information

S1 Text

Iterative Reweighed Least Squares. Consider the first order Taylor expansion

$$\begin{aligned} f_k(z_i;\boldsymbol{\beta}_k) &= \exp(\boldsymbol{\beta}_k^t\boldsymbol{w}_i) \\ &\approx \exp(\tilde{\boldsymbol{\beta}}_k^t\boldsymbol{w}_i) + \left.\frac{\partial f_k(z_i;\boldsymbol{\beta}_k)}{\partial\boldsymbol{\beta}_k}\right|_{\tilde{\boldsymbol{\beta}}_k}(\boldsymbol{\beta}_k-\tilde{\boldsymbol{\beta}}_k) \\ &\approx \exp(\tilde{\boldsymbol{\beta}}_k^t\boldsymbol{w}_i) + \exp(\tilde{\boldsymbol{\beta}}_k^t\boldsymbol{w}_i)\boldsymbol{w}_i^t(\boldsymbol{\beta}_k-\tilde{\boldsymbol{\beta}}_k) \\ &\approx (1-\boldsymbol{w}_i^t\tilde{\boldsymbol{\beta}}_k)\exp(\tilde{\boldsymbol{\beta}}_k^t\boldsymbol{w}_i) + \exp(\tilde{\boldsymbol{\beta}}_k^t\boldsymbol{w}_i)\boldsymbol{w}_i^t\boldsymbol{\beta}_k. \end{aligned}$$

Let $\boldsymbol{\lambda}_k^t = (f_k(z_1;\boldsymbol{\beta}_k),\ldots,f_k(z_n;\boldsymbol{\beta}_k))$ for which the Taylor expansion gives

$$\boldsymbol{\lambda}_k^t \approx \tilde{\boldsymbol{D}}_{1k}\tilde{\boldsymbol{\lambda}}_k + \tilde{\boldsymbol{D}}_{2k}\boldsymbol{W}\boldsymbol{\beta}_k,$$

with the matrices as defined in Section Bell-Shape Enriched Constrained Ordination analysis.

Upon introducing matrix notation in Equation 5 in Section Penalised Maximum Likelihood to replace the summation, and upon using the Taylor expansion, the estimating equation becomes approximately Equation 6 in Section Penalised Maximum Likelihood.
