# Supplementary material for: Constrained Ordination Analysis with Enrichment of Bell-Shaped Response Functions
Source: PLoS One. 2016 Apr 21;11(4):e0154079. doi: 10.1371/journal.pone.0154079 (PMC4839756; doi:10.1371/journal.pone.0154079)
Supplement: S1 Table — (PDF) [file pone.0154079.s002.pdf]

Supporting Information

S1 Table

Joint model fit.

Table 1. Comparison of the joint model fits for the first and second dimension from three ordination methods applied to the Antarctic lakes data. MSE gives the mean squared error calculated only among Bell-shaped species, MSE*stands for the mean squared error calculated from all species.

	BECOA	FCOA	CCA
$\sqrt{\text{MSE}}$	101.80	229.18	157.74
$\sqrt{\text{MSE}^*}$	152.68	152.98	157.74
