# Supplementary material for: Constrained Ordination Analysis with Enrichment of Bell-Shaped Response Functions
Source: PLoS One. 2016 Apr 21;11(4):e0154079. doi: 10.1371/journal.pone.0154079 (PMC4839756; doi:10.1371/journal.pone.0154079)
Supplement: S2 Text — (PDF) [file pone.0154079.s004.pdf]

Supporting Information

S2 Text

Newton-Raphson for the Maximisation of LR. The $\boldsymbol{\alpha}$ maximising Equitation 9 in Section Estimation of the Environmental Gradient is found by solving the equation

$$0 = \boldsymbol{U}(\boldsymbol{Y}, \boldsymbol{\alpha}) = \frac{\partial \log \mathrm{LR}(\boldsymbol{\alpha})}{\partial \boldsymbol{\alpha}} = \sum_{i=1}^{n} \sum_{k=1}^{s} \frac{\partial \log p_k(y_{ik} \mid z_i, \boldsymbol{\beta}_k)}{\partial \boldsymbol{\alpha}} - \sum_{i=1}^{n} \sum_{k=1}^{s} \frac{\partial \log p(y_{ik} \mid z_i, \boldsymbol{\beta})}{\partial \boldsymbol{\alpha}}. \quad (1)$$

The first term equals

$$\sum_{i=1}^{n} \sum_{k=1}^{s} (y_{ik} - \exp(\boldsymbol{\beta}_k^t \boldsymbol{w}_i)) \boldsymbol{A}_{ik}$$

with

$$\boldsymbol{A}_{ik} = \boldsymbol{\beta}_k^t \frac{\partial \boldsymbol{w}_i}{\partial z} \boldsymbol{x}_i.$$

Similarly, the second term of (1) can be written as

$$\sum_{i=1}^{n} \sum_{k=1}^{s} (y_{ik} - \exp(\boldsymbol{\beta}^t \boldsymbol{w}_i^*)) \boldsymbol{A}_i$$

with $\boldsymbol{A}_i = \boldsymbol{\beta}^t \frac{\partial \boldsymbol{w}_i^*}{\partial z} \boldsymbol{x}_i$ and in which we use $\boldsymbol{w}_i^*$ to stress that the structure of the common model may be different from the structure of the species-specific models (however, in most applications $\boldsymbol{w}_i^* = \boldsymbol{w}_i$).

Newton-Raphson requires the Hessian matrix

$$\begin{aligned} \boldsymbol{H}(\boldsymbol{Y}, \boldsymbol{\alpha}) &= \frac{\partial \boldsymbol{U}(\boldsymbol{Y}, \boldsymbol{\alpha})}{\partial \boldsymbol{\alpha}} \\ &= \sum_{i=1}^{n} \sum_{k=1}^{s} \big[-\exp(\boldsymbol{\beta}_k^t \boldsymbol{w}_i) \boldsymbol{A}_{ik} \boldsymbol{A}_{ik}^t + (y_{ik} - \exp(\boldsymbol{\beta}_k^t \boldsymbol{w}_i)) \boldsymbol{A}'_{ik} \\ &\quad + \exp(\boldsymbol{\beta}^t \boldsymbol{w}_i^*) \boldsymbol{A}_i^t \boldsymbol{A}_i - (y_{ik} - \exp(\boldsymbol{\beta}^t \boldsymbol{w}_i^*)) \boldsymbol{A}'_i \big], \end{aligned}$$

where

$$\boldsymbol{A}'_{ik} = \frac{\partial \boldsymbol{A}_{ik}}{\partial \boldsymbol{\alpha}} = \boldsymbol{\beta}_k^t \frac{\partial^2 \boldsymbol{w}_i}{\partial z^2} \boldsymbol{x}_i \boldsymbol{x}_i^t \text{ and } \boldsymbol{A}'_i = \frac{\partial \boldsymbol{A}_i}{\partial \boldsymbol{\alpha}} = \boldsymbol{\beta}^t \frac{\partial^2 \boldsymbol{w}_i^*}{\partial z^2} \boldsymbol{x}_i \boldsymbol{x}_i^t.$$

Newton-Raphson starts with an initial $\boldsymbol{\alpha}$, say $\boldsymbol{\alpha}^{(0)}$, which is updated by

$$\boldsymbol{\alpha}^{(m+1)} = \boldsymbol{\alpha}^{(m)} - \boldsymbol{H}^{-1}(\boldsymbol{Y}, \boldsymbol{\alpha}^{(m)}) \boldsymbol{U}(\boldsymbol{Y}, \boldsymbol{\alpha}^{(m)}), \quad (2)$$

$m = 0, 1, \ldots$ (until convergence).

Fisher scoring also requires the expectation of the Hessian, which becomes, upon using $\mathrm{E}\{Y_{ik} \mid z_i\} = \exp(\boldsymbol{\beta}_k^t \boldsymbol{w}_i)$,

$$\begin{aligned} \boldsymbol{J}(\boldsymbol{\alpha}) &= \mathrm{E}\{\boldsymbol{H}(\boldsymbol{Y}, \boldsymbol{\alpha}) \mid \boldsymbol{z}\} \\ &= \sum_{i=1}^{n} \sum_{k=1}^{s} \big[-\exp(\boldsymbol{\beta}_k^t \boldsymbol{w}_i) \boldsymbol{A}_{ik} \boldsymbol{A}_{ik}^t \\ &\quad + \exp(\boldsymbol{\beta}^t \boldsymbol{w}_i^*) \boldsymbol{A}_i^t \boldsymbol{A}_i - (\exp(\boldsymbol{\beta}_k^t \boldsymbol{w}_i) - \exp(\boldsymbol{\beta}^t \boldsymbol{w}_i^*)) \boldsymbol{A}'_i \big]. \end{aligned}$$

Newton-Raphson with fisher scoring replaces (2) with

$$\boldsymbol{\alpha}^{(m+1)} = \boldsymbol{\alpha}^{(m)} - \boldsymbol{J}^{-1}(\boldsymbol{\alpha}^{(m)})\boldsymbol{U}(\boldsymbol{Y}, \boldsymbol{\alpha}^{(m)}).$$
