# Supplementary material for: Constrained Ordination Analysis with Enrichment of Bell-Shaped Response Functions
Source: PLoS One. 2016 Apr 21;11(4):e0154079. doi: 10.1371/journal.pone.0154079 (PMC4839756; doi:10.1371/journal.pone.0154079)
Supplement: S3 Text — (PDF) [file pone.0154079.s005.pdf]

Supporting Information

S3 Text

Cross validation. In order to reduce the computational load, we propose a simplified cross-validation procedure in which the environmental gradient calculated from the complete data set, say $\hat{\boldsymbol{\alpha}}$, is kept constant throughout the calculations. In particular, the data set is randomly split into 10 equally large parts. We denote these subsets as $\boldsymbol{X}_j$ $(j = 1, \ldots, 10)$. The procedure now iterates over the following steps: in the jth step, the jth subset $\boldsymbol{X}_j$ is removed and the remaining 9 subsets, denoted by $\boldsymbol{X}_{(-j)}$, serve as the training data, from which the environmental scores $\boldsymbol{z}_{\text{train}} = \boldsymbol{X}_{(-j)}\hat{\boldsymbol{\alpha}}$ are computed, as well as the β parameter estimates from the penalized regression procedure, say $\hat{\boldsymbol{\beta}}_{-j}$. For the testing data the scores $\boldsymbol{z}_{\text{test}} = \boldsymbol{X}_{(j)}\hat{\boldsymbol{\alpha}}$ and, upon using $\hat{\boldsymbol{\beta}}_{-j}$, the predictions of the abundances are computed. The latter, and the observed abundances in the test data set are subsequently used for the calculation of the residuals. Finally, after completion of the 10 cross validation cycles, all residuals are squared and summed, resulting in the cross-validation estimate SSE. This procedure is repeated for a sequence of δs.

The cross-validated average LLR can be obtained analogously.
