# Supplementary material for: Constrained Ordination Analysis with Enrichment of Bell-Shaped Response Functions
Source: PLoS One. 2016 Apr 21;11(4):e0154079. doi: 10.1371/journal.pone.0154079 (PMC4839756; doi:10.1371/journal.pone.0154079)
Supplement: S4 Text — (PDF) [file pone.0154079.s006.pdf]

Supporting Information

S4 Text

Algorithm of absence/presence data. When the abundance data are replaced by the species absence ($Y_{ik} = 0$) / presence ($Y_{ik} > 0$) information, the probability of the presence of a species at certain location can be linked to environmental scores using the binomial distribution, and, for example, the logit link. In particular,

$$\pi_{ik} = \mathrm{P}\{Y_{ik} > 0 | z_i\} = \mathrm{expit}\left(a_k - \frac{(z_i - \mu_k)^2}{2t_k^2}\right).$$

The probability mass function is then written as

$$p_k(y_{ik} \mid z_i, \boldsymbol{\beta}_k) = \pi_{ik}^{\mathrm{I}[y_{ik}>0]}(1 - \pi_{ik})^{\mathrm{I}[y_{ik}=0]}.$$

For this model, the score equation for the penalised maximum likelihood estimator of parameter β_{jk} becomes,

$$\sum_{i=1}^{n} \frac{\partial \log p_k(\boldsymbol{\beta}_k \mid y_{ik}, z_i)}{\partial \beta_{jk}} = \sum_{i=1}^{n} \left(y_{ik} - \frac{\exp(\boldsymbol{\beta}_k^t \boldsymbol{w}_i)}{1 + \exp(\boldsymbol{\beta}_k^t \boldsymbol{w}_i)}\right) w_{ij} + n \frac{\partial \log g(\boldsymbol{\beta}_k)}{\partial \beta_{jk}} = 0. \quad (1)$$

Consider the first order Taylor expansion

$$\begin{aligned}
\mathrm{expit}(\boldsymbol{\beta}_k^t \boldsymbol{w}_i) &= \frac{\exp(\boldsymbol{\beta}_k^t \boldsymbol{w}_i)}{1 + \exp(\boldsymbol{\beta}_k^t \boldsymbol{w}_i)} \\
&\approx \frac{\exp(\tilde{\boldsymbol{\beta}}_k^t \boldsymbol{w}_i)}{1 + \exp(\tilde{\boldsymbol{\beta}}_k^t \boldsymbol{w}_i)} + \frac{\exp(\tilde{\boldsymbol{\beta}}_k^t \boldsymbol{w}_i)(1 + \exp(\tilde{\boldsymbol{\beta}}_k^t \boldsymbol{w}_i))\boldsymbol{w^t}_i - \exp^2(\tilde{\boldsymbol{\beta}}_k^t \boldsymbol{w}_i)\boldsymbol{w}_i^t}{(1 + \exp(\tilde{\boldsymbol{\beta}}_k^t \boldsymbol{w}_i))^2}(\boldsymbol{\beta}_k - \tilde{\boldsymbol{\beta}}_k) \\
&\approx \frac{\exp(\tilde{\boldsymbol{\beta}}_k^t \boldsymbol{w}_i)}{1 + \exp(\tilde{\boldsymbol{\beta}}_k^t \boldsymbol{w}_i)} + \left[\frac{\exp(\tilde{\boldsymbol{\beta}}_k^t \boldsymbol{w}_i)}{1 + \exp(\tilde{\boldsymbol{\beta}}_k^t \boldsymbol{w}_i)} - \left(\frac{\exp(\tilde{\boldsymbol{\beta}}_k^t \boldsymbol{w}_i)}{1 + \exp(\tilde{\boldsymbol{\beta}}_k^t \boldsymbol{w}_i)}\right)^2\right] \boldsymbol{w}_i^t \boldsymbol{\beta}_k \\
&\quad + \left[\left(\frac{\exp(\tilde{\boldsymbol{\beta}}_k^t \boldsymbol{w}_i)}{1 + \exp(\tilde{\boldsymbol{\beta}}_k^t \boldsymbol{w}_i)}\right)^2 - \frac{\exp(\tilde{\boldsymbol{\beta}}_k^t \boldsymbol{w}_i)}{1 + \exp(\tilde{\boldsymbol{\beta}}_k^t \boldsymbol{w}_i)}\right] \boldsymbol{w}_i^t \tilde{\boldsymbol{\beta}}_k.
\end{aligned}$$

Let $\boldsymbol{\theta}_k^t = (\mathrm{expit}(\boldsymbol{\beta}_k^t \boldsymbol{w}_1), \ldots, \mathrm{expit}(\boldsymbol{\beta}_k^t \boldsymbol{w}_n))$ for which this Taylor expension gives

$$\boldsymbol{\theta}_k^t \approx \tilde{\boldsymbol{\theta}}_k + (\tilde{\boldsymbol{D}}_3 - \tilde{\boldsymbol{D}}_3^2)\boldsymbol{W}\boldsymbol{\beta}_k - (\tilde{\boldsymbol{D}}_3 - \tilde{\boldsymbol{D}}_3^2)\boldsymbol{W}\tilde{\boldsymbol{\beta}}_k,$$

where

$$\tilde{\boldsymbol{\theta}}_k = \mathrm{expit}(\boldsymbol{W}\tilde{\boldsymbol{\beta}}_k) \quad \tilde{\boldsymbol{D}}_3 = \mathrm{Diag}(\mathrm{expit}(\boldsymbol{\beta}_k^t \boldsymbol{w}_i))$$

and $\boldsymbol{W}$ is defined as in the paper. Given a $\tilde{\boldsymbol{\beta}}_k$, Equation (1) gives a closed form for an updated estimate,

$$\hat{\boldsymbol{\beta}}_k = \left(\boldsymbol{W}^t(\tilde{\boldsymbol{D}}_3 - \tilde{\boldsymbol{D}}_3^2)\boldsymbol{W} + n\gamma \boldsymbol{D}^{-1}\right)^{-1} \left[(\boldsymbol{Y} - \tilde{\boldsymbol{\theta}}_k)^t \boldsymbol{W} + \tilde{\boldsymbol{\beta}}_k^t \boldsymbol{W}^t (\tilde{\boldsymbol{D}}_3 - \tilde{\boldsymbol{D}}_3^2)\boldsymbol{W} + n\gamma \boldsymbol{\delta}^t \boldsymbol{D}^{-1}\right],$$

in which the terms $n\gamma\boldsymbol{D}^{-1}$ and $n\gamma\boldsymbol{\delta}^t\boldsymbol{D}^{-1}$ arise from the penalisation (see also algorithm 1 in Section Penalised Maximum Likelihood).
