# Supplementary material for: Constrained Ordination Analysis with Enrichment of Bell-Shaped Response Functions
Source: PLoS One. 2016 Apr 21;11(4):e0154079. doi: 10.1371/journal.pone.0154079 (PMC4839756; doi:10.1371/journal.pone.0154079)
Supplement: S5 Text — (PDF) [file pone.0154079.s007.pdf]

Supporting Information

S5 Text

Implementation in R.

```
library(MASS)
IRPMLE<-function(y1,z,ga,del,sd,control.value,...){
n<-length(y1)
w<-cbind(rep(1,n),z,z^2)
beta.start<-glm(y1~z+I(z^2),family=poisson)$coefficients
d.inverse<-matrix(0,3,3)
diag(d.inverse)<-c(0,0,1/(sd^2))
iter<-1
continue<-TRUE
d1.tilde<-d2.tilde<-matrix(0,ncol=n,nrow=n)
while(continue){
#print(beta.start)
    temp<-w%*%beta.start
lambda.tilde<-exp(temp)
diag(d1.tilde)<-1-temp
diag(d2.tilde)<-lambda.tilde
p1<-t(w)%*%d2.tilde%*%w+n*ga*d.inverse
p2<-t(y1-d1.tilde%*%lambda.tilde)%*%w+n*ga*t(del)%*%d.inverse
p2<-as.vector(p2)
 beta.new<-tryCatch({solve(p1, p2)},error=function(e) {return(NA)})
        if(is.na(beta.new))
        {
        gen.inv<-ginv(p1)
        beta.new<-gen.inv%*%p2
        }

diff<-sum((beta.new-beta.start)^2)
continue<-(diff>control.value&&iter<50)
iter<-iter+1
beta.start<-beta.new
}
return(beta.start)
}
#####################
newNR_pMLE <- function(sp,alpha,envar,ga,del,MLE=c('pMLE','IRPMLE'),sd=1,
control.value=1e-3,steptol=1e-3,Newton=TRUE,...)
{
    nc <- ncol(sp)
    nr <- nrow(sp)
    alpha <- alpha / sqrt(sum(alpha^2))
    wt <- colSums(sp) / sum(sp)
    sp.all <- rowSums(wt*sp)
    envar <- as.matrix(envar)
```

```
  iter <- 1
  continue <- TRUE


  while(continue)
  {
      z <- envar %*% alpha
      z<-scale(z)
      w <- cbind(rep(1, nr), z, z^2)
      dw <- cbind(rep(0, nr), rep(1, nr), 2*z)
      ddw <- cbind(rep(0, nr), rep(0, nr), rep(2, nr))

beta.new<-switch(MLE,'pMLE'=apply(sp,2,function(y)
pMLE(y1=y,z=z,ga=ga,del=del,sd=sd,control.value=control.value)),
 'IRPMLE'=apply(sp,2,function(y) IRPMLE(y1=y,z=z,ga=ga,del=c(0,0,del),
 sd=sd,control.value=control.value)))

      beta.new <- as.matrix(beta.new)

      beta.all <- glm(sp.all ~ z + I(z^2), family = poisson)$coefficients
      A1 <- dw %*% beta.new
      fit.res <- sp-exp(w %*% beta.new)
      A2 <- dw %*% beta.all
      fit1 <- exp(w %*% beta.new)

      fit.all <- exp(w %*% beta.all)
      A1.prime <- ddw %*% beta.new
      A2.prime <- ddw %*% beta.all
      ncx <- ncol(envar)

      score1<-score2<-score<-c()

      ###
      ### FIRST VECTORISATION: computing *scores*
      ###
      res1 <- sapply(seq_len(ncx),
              FUN = function(i, a, e, fit)
              {
                  sum(a * e[, i] * fit)
              },
              a = A1, e = envar, fit = fit.res)

      res2 <- sapply(seq_len(ncx),
              FUN = function(i, a, e, fit, s, n)
              {
                  tmp <- a * e[, i]
                  sum(tmp * s) - sum(tmp * fit) * n
              },
              a = A2, e = envar, fit = fit.all, s = rowSums(sp), n = nc)
      score <- res1 - res2
```

```
hessian <- matrix(NA, nrow = ncx, ncol = ncx)

###
### SECOND VECTORISATION: computing *hessian*
###
listHess <- sapply(seq_len(ncx),
        FUN = function(i, e, ...)
        {
            sapply(seq_len(ncx),
                    FUN = function(i, a1, a2, e1, e2,
                                          a1Prime, a2Prime,
                            fit1, fitR, fitA, n, s,Newton,rsf)
                    {
                    p1 <- sum(-fit1 * a1 * e1[, i] * e2)
                    p3 <- sum(fitA * a2 * e1[, i] * e2) * n
                      if(Newton){

                     p2 <- sum(fitR * a1Prime * e1[, i] * e2)
                     p4 <- sum(fitA * a2Prime * e1[, i] * e2) * n -
                                sum(a2Prime * e1[, i] * e2 * s)
                      p1+p2+p3+p4}

                      else {
                       p5<-sum(fitA*a2Prime*e1[,i]*e2)*n
                         -sum(a2Prime*e1[,i]*e2*rsf)
                        p1+p3+p5
                         }

                    },
                 a1 = A1^2, a2 = A2^2, e1 = envar, e2 = envar[, i],
                 a1Prime = A1.prime, a2Prime = A2.prime,
                 fit1 = fit1, fitR = fit.res, fitA = fit.all,
                 s = rowSums(sp), n = nc,rsf=rowSums(fit1),
                Newton=Newton)
        }, e = envar,
        A1 = A1, A2 = A2, envar = envar,
        A1.prime = A1.prime, A2.prime = A2.prime,
        fit1 = fit1, fit.res = fit.res, fit.all = fit.all,
        sp = sp, nc = nc,Newton=Newton)

inv<-tryCatch({solve(listHess, score)},
           error=function(e) {return(NA)})
if(is.na(inv))
{
gen.inv<-ginv(listHess)
alpha.new<-alpha-gen.inv%*%score
}
else
alpha.new <- alpha-inv
#- indeed
alpha.new<-alpha.new/sqrt(sum(alpha.new^2))
```

```
        diff <- sqrt(sum((alpha.new-alpha)^2))/sqrt(sum(alpha^2))
        continue <- (diff>steptol&&iter<1000)
        alpha <- round(alpha.new, digits = 4)
        #print(alpha)
        #result[[iter]]<-alpha
        iter <- iter+1
    }
    z.final<- envar %*% alpha
    beta.final<-apply(sp,2,function(y) IRPMLE(y1=y,z=z.final,
                    ga=ga,del=c(0,0,del),sd=sd,
                    control.value=control.value))
 w.final <- cbind(rep(1, nr), z.final, z.final^2)
 fitted.value <-fitted.log<- exp(w.final %*% beta.final)
 fitted.log<-log(fitted.value[fitted.value!=0])
 fit.all.final<-fit.all.log<-glm.fit(w.final,sp.all,
                    family=poisson())$fitted.value
 fit.all.log<-log(fit.all.final[fit.all.final!=0])

    lr<- (sum(sp*fitted.log-fitted.value)
    - sum((beta.final[3,]-del)^2/sd)*nr
    -sum(fit.all.log*rowSums(sp))
    +sum(fit.all.final)*nc)

    lr.sp<-colSums(sp*fitted.log-fitted.value)-t(fit.all.log)

    sse<-colSums((sp-fitted.value)^2)/nr
    return(list('alpha'=alpha,'coefficients'=beta.final,
         'inter.num'=iter,'z'=z.final,'lr'=lr,'sse'=sse,'lr.sp'=lr.sp))
}

###############
# The BECOA
###############
newNR_pMLE(sp, alpha, envar, ga, del, MLE='IRPMLE')
#sp: abundance matrix
#alpha: the initial coefficient
# envar: environmental matrix
# del: value of the penalisation parameter
# MLE: penalized maximum likelihood algorithm: 'pMLE'
#stands for the algorithm 1and 'IRPMLE' stands
#for the algorithm 2 in the paper.
library(VGAM)
data(hspider)#load the hunting spider data
env<-as.matrix(hspider[,1:6])#enviromental data matrix
dat<-as.matrix(hspider[,7:18])#spider abundance matrix

becoa<-newNR_pMLE(sp = dat, alpha = rnorm(6),
  envar = env, ga=1,del = -0.7,MLE='IRPMLE')
#result attributes
#alpha: the estiamted coefficients
#coefficients: the estiamted regression coefficients
```

```
#inter.num: number of iteration
#z: environmental gradient
#lr: the likelihood ratio value of all species
#sse: the sum of square error for each species
#lr.sp the likelihood value of the model fit for each species.
```
